# The Methylation Inhibitor 5-Aza-2′-Deoxycytidine Induces Genome-Wide Hypomethylation in Rice

**DOI:** 10.1186/s12284-022-00580-6

**Published:** 2022-07-02

**Authors:** Shuo Liu, Yu Bao, Hui Deng, Guanqing Liu, Yangshuo Han, Yuechao Wu, Tao Zhang, Chen Chen

**Affiliations:** 1 Jiangsu Key Laboratory of Crop Genomics and Molecular Breeding/Jiangsu Key Laboratory of Crop Genetics and Physiology, Agricultural College of Yangzhou University, Yangzhou, 225009 China; 2 Jiangsu Co-Innovation Center for Modern Production Technology of Grain Crops, Yangzhou University, Yangzhou, 225009 China; 3 Key Laboratory of Plant Functional Genomics of the Ministry of Education/Joint International Research Laboratory of Agriculture and Agri-Product Safety, The Ministry of Education of China, Yangzhou University, Yangzhou, 225009 China

**Keywords:** Rice, Methylation, 5-Aza-2′-deoxycytidine, Transposable element, RdDM

## Abstract

DNA methylation is a conserved epigenetic modification which is vital for regulating gene expression and maintaining genome stability in both mammals and plants. Homozygous mutation of rice *methyltransferase 1* (*met1*) gene can cause host death in rice, making it difficult to obtain plant material needed for hypomethylation research. To circumvent this challenge, the methylation inhibitor, 5-Aza-2′-deoxycytidine (AzaD), is used as a cytosine nucleoside analogue to reduce genome wide hypomethylation and is widely used in hypomethylation research. However, how AzaD affects plant methylation profiles at the genome scale is largely unknown. Here, we treated rice seedlings with AzaD and compared the AzaD treatment with *osmet1-2* mutants, illustrating that there are similar CG hypomethylation and distribution throughout the whole genome. Along with global methylation loss class I transposable elements (TEs) which are farther from genes compared with class II TEs, were more significantly activated, and the RNA-directed DNA Methylation (RdDM) pathway was activated in specific genomic regions to compensate for severe CG loss. Overall, our results suggest that AzaD is an effective DNA methylation inhibitor that can influence genome wide methylation and cause a series of epigenetic variations.

**Supplementary Information:**

The online version contains supplementary material available at 10.1186/s12284-022-00580-6.

## Background

DNA methylation occurs predominantly at CG sites in mammals, but in plants occurs at three different sequence contexts being symmetrical CG, CHG, and asymmetrical CHH sites (H = A, T, or C) (Ji et al. 2018; Kenchanmane Raju et al. 2019). CG methylation is the most common and predominant type as it is found across all eukaryotes. The maintenance of CG methylation relies on the capacity to transfer methylation to cytosines on the opposite DNA strand during replication (Gruenbaum et al. 1981). This process of methylation transfer is accomplished by DNA methyltransferase 1 (DNMT1) in mammals and its ortholog methyltransferase 1 (MET1) in plants (Kenchanmane Raju et al. 2019). CHG methylation is similar to CG methylation in that it is also symmetrical (Gruenbaum et al. 1981), but maintained by a different enzyme, chromomethylase 3 (CMT3) (Lindroth et al. 2001; Papa et al. 2001). CHH methylation differs from CG and CHG methylation in that there is not a mirrored cytosine on the opposite DNA strand (Meyer et al. 1994; Griffin et al. 2016), and as such CHH methylation can only be established de novo as opposed to being maintained like CG and CHG methylation. One way to establish de novo CHH methylation in H3K9me2 regions is through another member of the CMT family, CMT2 (Kenchanmane Raju et al. 2019). RdDM is another pathway that can establish methylation in all three contexts via domains rearranged methylase 2 (DRM2), which is directed by 24-nt siRNAs. RdDM is in particular associated with high levels of CHH methylation (Niederhuth and Schmitz 2017).

DNA demethylation is closely related to plant growth and development, as well as vernalization and flowering. Since the establishment and maintenance of methylation involve different enzymes and pathways, different strategies can be used to obtain demethylated plants, such as constructing different methyltransferase mutants and treating plants with methylation inhibitors to acquire plant material with low methylation levels. In plants, CG methylation is maintained by MET1, the orthologue of mammalian DNMT1. The *met1* mutant is widely used for research purposes in *Arabidopsis thaliana* and rice plants (Kankel et al. 2003). There are two *MET1* homologs, *OsMET1-1* and *OsMET1-2*, encoded by the rice genome. The *osmet1-2* homozygous mutation is lethal to rice seedlings and difficult to obtain (Hu et al. 2014), whereas the *osmet1-1* mutation exhibits limited impact on DNA methylation. 5-Aza-2′-deoxycytidine (AzaD) and 5-azacytidine (5-AZA) are two cytosine nucleoside analogues having the same function that substitute DNA and covalently trap the methyltransferase to deplete its activity, thereby causing demethylation mainly in the CG context (Christman. 2002). AzaD was originally widely used in cancer treatments (Christman 2002; Gomyo et al. 2004) and has recently been used as a DNA methylation inhibitor to study plant phenotypic abnormalities caused by methylation deficiency. In *Cephalotaxus mannii*, AzaD was found to improve enzyme activity associated with the metabolic process of cell growth and product biosynthesis (Wei et al. 2019). AzaD enhances cold resistance and expression of cold-inducible genes in *Arabidopsis thaliana* (Song et al. 2017). 5-AZA was compared to another methylation inhibitor, zebularine (ZEB), and was found to cause similar demethylation patterns as ZEB but was more effective at higher concentrations in *Arabidopsis* (Griffin et al. 2016). While AzaD and 5-AZA are widely used chemical inhibitors in plant phenotypic studies, little is known about the specific impacts of AzaD on the methylation level of the whole genome and how the plant responds to AzaD treatment.

In the present study, rice seedlings were treated with AzaD and methylation loss across the whole genome was examined. The methylation levels of the rice genome of all three contexts significantly declined after AzaD treatment. The distribution patterns of demethylation and differentially methylated regions (DMRs) were similar to *osmet1-2* mutant to a certain extent, but not exactly the same. After AzaD treatment, rice development was delayed and genes involved in plant development were affected. Additionally, we describe the relationship between TE, CHH, and siRNAs after AzaD treatment. In summary, we demonstrated, in detail, the molecular response of rice DNA methylation to AzaD, filling an important knowledge gap surrounding plant DNA demethylation.

## Results

### Methylation Status was Significantly Reduced After AzaD Treatment

**Fig. 1:**
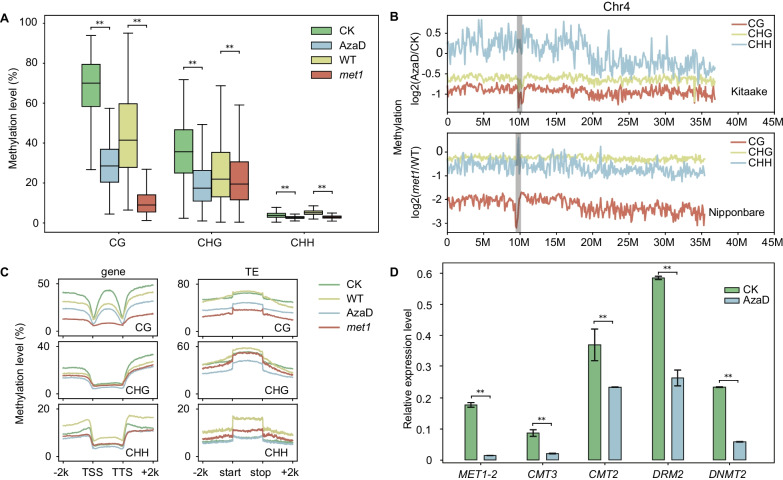
Methylation status was significantly reduced after AzaD treatment. **a** Boxplot of global methylation levels of CG, CHG, and CHH methylation in AzaD treated and *osmet1-2* mutant rice. Methylation levels of the three contexts showed significant reductions in both AzaD treated and *osmet1-2* rice. **b** Fold changes of methylation level for CG, CHG, and CHH methylation on chromosome 4. AzaD treatment induced moderate CHH methylation increases in the heterochromatin region. The grey boxes mark the centromere regions. **c** Metaplot of methylation levels in genes, TE bodies and their flanking regions. **d** Relative expression levels of selected methyltransferases. Transcript levels of *MET1-2*, *CMT3*, *CMT2*, *DRM2*, and *DNMT2* were significantly reduced after AzaD treatment. *p < 0.05, **p < 0.01 by Student’s t-test. CK and WT refer to check (CK, Kitaake without AzaD treatment) and WT (wild-type Nipponbare without mutation of *MET1-2*) plants, respectively

To assess the impact of AzaD on rice DNA methylation, we treated Kitaake (*Oryza sativa* ssp. *geng/japonica*) rice seedlings with AzaD and performed whole-genome bisulfite sequencing (WGBS). The results of WGBS revealed a global reduction of CG, CHG, and CHH methylation (Fig. 1A). Among the three contexts, CG methylation experienced the most dramatic decline (57.6%), from 68.4% in Kitaake to 29.0% in AzaD treated plants in average. This reduction was of a lesser magnitude than that of *osmet1-2* with respect to its wild-type (WT) Nipponbare (*Oryza sativa* ssp. *geng/japonica*) plants, in which average CG methylation level was reduced by 77.4%, from 44.7% in the WT to 10.1% in *osmet1-2* mutant plants (Hu et al. 2014) (Fig. 1A). Compared with CG methylation, AzaD treatment had a lesser effect on CHG and CHH methylation, but these were also significantly reduced (CHG: from 36.2% to 19.3%; CHH: from 3.9 to 2.8%). In addition, the decrease of CHH methylation in AzaD treated plants was lower than that in *osmet1-2* (CHH from 5.21 to 2.98%), and conversely, the decrease of CHG methylation in AzaD treated plants was significantly greater than that in *osmet1-2* mutants (25.8 to 22.1%) (Fig. 1A).

To better understand the effects of AzaD treatment, we measured changes in DNA methylation levels between AzaD treated and control plants (Fig. 1B, Additional file 1: Fig. S1 and Additional file 1: Fig. S2). The results suggested that after AzaD treatment, the loss of CG and CHG methylation was relatively more prevalent throughout the whole chromosome and showed no distinct preference for heterochromatin or euchromatin (Fig. 1B, Additional file 1: Fig. S1 and Additional file 1: Fig. S2). On the contrary, CHH methylation levels were slightly elevated in heterochromatin regions and declined in euchromatin regions. Compared to AzaD treatment, *osmet1-2* mutants showed less CHG methylation decline, and CHH methylation was reduced throughout the whole chromosome (Fig. 1B, Additional file 1: Fig. S1 and Additional file 1: Fig. S2). The reduction of methylation levels in heterochromatin regions was milder than in euchromatin regions in *osmet1-2*, but methylation levels became evenly reduced after AzaD treatment (Fig. 1B, Additional file 1: Fig. S1 and Additional file 1: Fig. S2). The methylation levels of genes and TEs were also examined, respectively (Fig. 1C). CG methylation levels declined more severely in gene bodies and TEs of *osmet1-2* mutants than AzaD treated plants. However, in CHG methylation an opposite pattern was observed, in which the extent of methylation reduction was greater in the AzaD treatment than in *osmet1-2*, both in genes and TEs, with *osmet1-2* methylation levels remaining slightly reduced in gene bodies (Fig. 1C). CHH methylation levels of both genes and TEs experienced little change in AzaD treated plants, but was significantly reduced in *osmet1-2* mutants (Fig. 1C). The difference in CHG and CHH methylation level changes between the AzaD treatment and *osmet1-2* mutant plants suggested that AzaD treatment and mutation of *OsMET1-2* may affect distinct pathways involved in methylation establishment and maintenance. Real-time quantitative PCR (RT-qPCR) results showed that the expression levels of the primary methyltransferases were significantly down-regulated, which is in correspondence with the whole genome demethylation (Fig. 1D). *CMT3* was up-regulated in *osmet1-2* mutants, which explains the more severe loss of CHG methylation in AzaD treated plants in comparison with *osmet1-2* mutants (Hu et al. 2014).

### DMR Identification and Comparison with *osmet1-2* Mutants

**Fig. 2:**
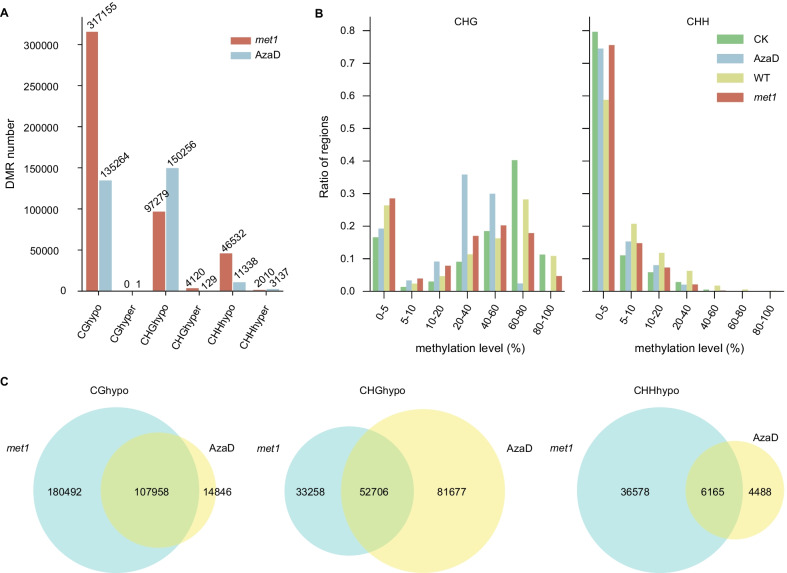
Identified DMRs in AzaD treated and *osmet1-2* mutant rice. **a** Number of identified DMRs. DMRs in AzaD treated rice were identified relative to the Kitaake cultivar (CK). DMRs in *osmet1-2* mutants were identified relative to the Nipponbare cultivar (WT). **b** Frequency distribution of CHG and CHH methylation levels in CG hypo DMRs. The loss of CG methylation was accompanied by the loss of CHG and CHH methylation. **c** Venn diagrams show the DMR overlap ratio between AzaD treated and *osmet1-2* mutant rice. DMRs located in the collinear regions identified between genomes of Kitaake and Nipponbare rice cultivars were calculated

With the aim of studying methylation changes more intuitively, DMRs in AzaD treated and *osmet1-2* mutant plants were identified with respect to the check (CK, Kitaake without AzaD treatment) and WT (wild-type Nipponbare) plants, respectively, according to specific standards for each methylation context (Fig. 2A). DMRs was determined when the differences in methylation level reach 40% for CG, 20% for CHG, and 10% for CHH (Higo et al. 2020; Shi et al. 2021), respectively. 135,264 CG hypo DMRs were identified in AzaD treated plants. There were almost no CG hyper DMRs identified after AzaD treatment. Even more CHG hypo DMRs (150,256) were identified after AzaD treatment, due to the different standards used between CG and CHG DMR identification. Moreover, comparing to CG and CHG, fewer CHH DMRs were identified: 11,338 CHH hypo DMRs and 3137 CHH hyper DMRs (Fig. 2A).

The number of CG hypo DMRs in AzaD treated plants, for which 135,264 CG hypo DMRs were identified, was lower than that in *osmet1-2* mutants, indicating a more moderate impact of AzaD treatment on CG methylation than *osmet1-2* mutation. There was a more extensive CHG reduction and more CHG hyper DMRs in the AzaD treated plants than in *osmet1-2*. Most of the CHG hypo DMRs in AzaD treated plants have the CHG methylation difference between 20 and 40%. There were only 17,123 CHG hypo DMRs in AzaD treated plants with CHG methylation difference more than 40%, but the number was still larger than that in *met1-2* mutants (9455 CHG hypo DMRs with CHG methylation difference more than 40%), which means CHG methylation was affected more heavily in AzaD treated plants than in *met1-2* mutants. According to the qRT-PCR result, *CMT3*, which was responsible for CHG maintenance, was up-regulated in met1-2 mutants, but was significantly down-regulated in AzaD treated rice (Fig. 1D) (Hu et al. 2014). Interestingly, a higher proportion of CHH hyper DMRs were found in AzaD treated plants (AzaD treated rice: 21.7%, *osmet1-2* mutants: 4.1%) (Fig. 2A).

The mutation of *OsMET1-2* mainly affected CG methylation, but according to previous studies, CHG and CHH methylation were reduced as well if CG methylation is first depleted in the same region (Ji et al. 2018). A frequency distribution of CHG and CHH methylation levels in CG hypo DMRs was created to determine if AzaD treatment would cause CHG and CHH methylation reductions in CG hypo DMRs (Fig. 2B). The results showed that in the CG hypo DMRs, CHG methylation was down-regulated in the AzaD treated plants, consistent with *osmet1-2* mutants. After AzaD treatment, the number of CG hypo DMRs with CHG levels greater than 60% was reduced more than in *osmet1-2* mutants, indicating a greater reduction of CHG methylation resulting from AzaD treatment. Surprisingly, the CHH level in CG hypo DMRs was not completely down-regulated. The number of CG hypo DMRs with CHH methylation levels of 5–20% was increased, suggesting that in specific CG hypo DMRs, CHH methylation was activated, which was not observed in *osmet1-2* mutants (Fig. 2B).

The distribution of DMRs in AzaD treated and *osmet1-2* plants are quite similar in many aspects except for some key distinctions (Additional file 1: Figure S3). Each kind of DMR shared a similar distribution ratio in the 1 kb regions upstream and downstream of genes, with the highest ratio being that of CHH hypo DMRs (CG hypo: 11% in AzaD, 13% in *osmet1-2*; CHG hyper: 15% in AzaD, 20% in *osmet1-2*; CHG hypo: 12% in AzaD, 15% in *osmet1-2*; CHH hyper: 13% in AzaD, 16% in *osmet1-2*; CHH hypo: 36% in AzaD, 33% in *osmet1-2*). Additionally, CG and CHG DMRs distributed in TEs also shared similarities (CG hypo: 18% in AzaD, 17% in *osmet1-2*; CHG hyper: 9% in AzaD, 7% in *osmet1-2*; CHG hypo: 23% in AzaD, 25% in *osmet1-2*). A similar pattern was also observed in CHH hypo DMRs within genes, between AzaD treated and *osmet1-2* plants (CHH hypo: 17% in AzaD, 15% in *osmet1-2*). Moreover, variations in distribution ratios were also found between the AzaD treatment and *osmet1-2*. More CG and CHG hypo DMRs in *osmet1-2* were found to locate within genes (CG hypo: 20% in AzaD, 34% in *osmet1-2*; CHG hypo: 7% in AzaD, 21% in *osmet1-2*), while the number of CHH hyper DMRs located in TEs was larger in AzaD treated plants than that in *osmet1-2* (CHH hyper: 15% in AzaD, 7% in *osmet1-2*) (Additional file 1: Figure S3). This indicates that AzaD treatment may affect different methylation processes compared to mutation of *OsMET1-2*.

To further compare the similarities and differences between AzaD treated and *osmet1-2* plants, we identified syntenic regions between the Nipponbare and Kitaake rice cultivar genomes and compared the overlap ratio between DMRs by judging whether the DMR corresponding regions in the two backgrounds overlapped (Fig. 2C). As a result, CG hypo DMR showed the highest overlap ratio (87.91%) between AzaD treated plants and *osmet1-2* mutants (Fig. 2C), suggesting high consistency in the area affected by AzaD treatment and *osmet1-2* mutation on CG context. In addition, CHG and CHH hypo DMRs showed moderate overlap levels, with overlap ratios of 39.22% and 57.87%, respectively (Fig. 2C). However, CHG and CHH hyper DMRs had overlap ratios of only 2.56% and 6.88%, respectively (Additional file 1: Figure S4). The high overlap ratio of hypo DMRs indicated a similar demethylation pattern.

In all, though AzaD treatment primarily caused demethylation in the CG context, CHG and CHH methylation levels were significantly decreased as well. Comparative analysis with *osmet1-2* mutants also revealed similarities in the CG methylome between the AzaD treated plants and *osmet1-2* mutants. These results suggest that AzaD treatment in rice can mimic *osmet1-2* mutation to resolve the difficulty of obtaining CG demethylated materials.

### TEs were Significantly Activated According to Distance from Genes

**Fig. 3:**
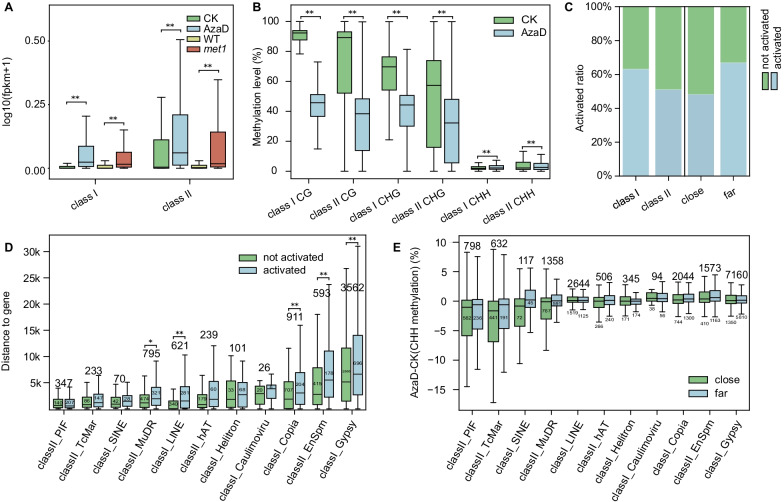
TEs were significantly activated according to distance from genes. **a** TE expression levels in AzaD treated and *osmet1-2* mutant rice. Class I retrotransposons and class II DNA transposons were significantly up-regulated in AzaD treated and *osmet1-2* mutant rice. **b** CG, CHG, and CHH methylation levels of class I retrotransposons and class II DNA transposons in CK and AzaD treated rice. **c** Activated ratio of class I and class II TEs (left), close (distance to the nearest gene less than 2 kb) and far (distance to the nearest gene more than 2 kb) TEs (right). TEs with log2FoldChange(FPKM + 1) > 2 were defined as activated TEs. Class I and far TEs had higher activation ratios. **d** Distances of activated and not activated TEs from different TE families to the nearest genes. TE families were ordered according to the average distance to the closest gene. Activated TEs tended to be farther from genes. **e** CHH methylation level changes of close and far TEs from different families. TE families were ordered according to the average distance to closest gene. TEs in families farther from genes showed increased CHH methylation levels. *p < 0.05, **p < 0.01 by Student’s t-test

TEs are an important part of the genome and have the ability to move from one location in the genome to another (Lanciano and Cristofari 2020). DNA methylation is a primary method used to suppress transposon expression. Although methylation levels were reduced both in the AzaD treated and *osmet1-2* plants, overall gene expression levels showed no significant elevation (Additional file 1: Figure S5). However, TEs in AzaD treated and *osmet1-2* plants both showed significant activation (Fig. 3A), which is in accordance with the observation that TE expression is mainly affected by methylation levels (Deniz et al. 2019; Ewing et al. 2020; Wambui Mbichi et al. 2020). Further experiments were carried out to reveal how TEs respond to AzaD treatment.

TEs are often separated into two major classes: class I TEs, also called retrotransposons, utilize an RNA intermediate that is reverse transcribed before genomic reinsertion; class II TEs, or DNA transposons, move via excision from one location in the genome followed by insertion into another (Capy et al. 1997; Takata et al. 2007). An inherent difference in methylation levels was found in CK plants between class I retrotransposons and class II DNA transposons. CG and CHG methylation levels in class I retrotransposons are higher than that in class II DNA transposons (Fig. 3B) and both were down-regulated in AzaD treated plants. Contrastingly, CHH methylation levels were higher in class II DNA transposons than in class I retrotransposons. Interestingly, after AzaD treatment, CHH methylation levels were increased in class I retrotransposons, while CHH methylation of class I TEs in *osmet1-2* plant were significantly down-regulated (Additional file 1: Figure S6). In addition, class II DNA transposons were demethylated in the CHH context, which was concordant with that happened in CG and CHG contexts. (Fig. 3B). This was accompanied with preferential distribution of class I TEs in heterochromatin, explaining the CHH hyper methylation in heterochromatin (Additional file 1: Figure S7). Demethylation may cause TE activation because of the negative association between methylation and TE expression. We characterized the effects of AzaD-induced methylome changes on TE expression. The results showed that 5118 of 19,788 TEs (22.53%) showed significantly different expression levels after AzaD treatment, of which 4459 TEs were up-regulated (log2FoldChange(FPKM + 1) > 1) and 659 TEs were down-regulated (log2FoldChange(FPKM + 1) < −1).

We further compared the response of the two classes of TE to AzaD treatment. Some ‘dead’ TEs were removed as they weren’t expressed both in CK and AzaD treated rice, while the remaining TEs consisted of 5,291 live class I retrotransposons and 2,207 class II DNA transposons. The CG and CHG methylation level in activated TEs (log2FoldChange(FPKM + 1) > 2) experienced significantly more severe loss than TEs not activated (Additional file 1: Figure S8). But the CHH methylation level in activated TEs was down-regulated less than not activated TEs because the compensation in CHH methylation, which will be described later. After AzaD treatment, 63.03% of live class I TEs was activated (log2FoldChange(FPKM + 1) > 2), which was significantly higher than that of class II TEs activated (50.92%) (Fisher’s exact test, *p* < 3.84 × 10^–58^) (Fig. 3C left). This indicates that class I TE expression is more sensitive to AzaD treatment than that of class II TEs. The different methylation changes between class I and II TEs brings into question the essential reason for this phenomenon and whether more undetermined differences between class I retrotransposons and class II DNA transposons exist.

TE expression patterns were found to be relevant to their distance to the closest gene, with class I TEs being distributed more in heterochromatin regions (Rebollo et al. 2011; Eichten et al. 2012), so we hypothesized that the difference in activating ratios of the two TE classes was related to their distance from genes. To confirm this hypothesis, TEs were classified into two groups: close (distance to closest gene less than 2 kb) and far (distance to closest gene more than 2 kb), and significantly higher activating ratios were found in the far TEs (Fisher’s exact test, *p* < 4.01 × 10^–22^) (Fig. 3C right). More precisely, TEs were classified into further groups according to their superfamilies and sorted by their average distance to the closest gene (Additional file 1: Figure S9, Fig. 3D). In each group, activated TEs had a farther distance from the closest gene (Fig. 3D), the discovery of which further confirmed our previous hypothesis that the activating ratio of the two TE classes was due to their distance from genes.

After classification into different families, TEs from different families showed divergent methylation patterns. Short interspersed nuclear elements (SINEs), belonging to class I retrotransposons, showed the highest CHH methylation levels among all class I retrotransposons (Zhang et al. 2015), which was also illustrated in our data (Additional file 1: Figure S10). We speculated the high CHH methylation levels in SINEs may also be associated with their distance to the nearest genes. To explain the phenomenon of SINEs having abnormally higher CHH methylation levels, the methylation level of each TE family was calculated to reveal correlations between TE methylation levels and distance to the nearest gene. TE families farther from genes showed higher CG and CHG methylation levels (Additional file 1: Figure S10A, B). An opposite pattern was observed for CHH methylation levels, with more distant TEs exhibiting relatively lower CHH methylation levels (Additional file 1: Figure S10C). SINEs, as a family from class I retrotransposons, were closer to genes unlike other families of class I TEs (Additional file 1: Figure S10C). This abnormal distance to genes led to their higher CHH methylation levels than other families of class I retrotransposons. The correlation between methylation changes and distance to the nearest gene of each TE was also found after AzaD treatment. Overall, CG methylation levels decreased more severely in more distant TEs (Additional file 1: Figure S11), which might be the reason for activation of these TEs. Conversely, more distant TEs exhibited CHH hypermethylation after AzaD treatment (Fig. 3E). A similar pattern in CG methylation also happened in *osmet1-2* mutants (Additional file 1: Figure S11), while the CHH hyper methylation was not found in *osmet1-2* mutants (Additional file 1: Figure S12).

### 24-nt siRNAs Involved in the RdDM Pathway were Altered as a Rapid Response to CG Methylation loss

**Fig. 4:**
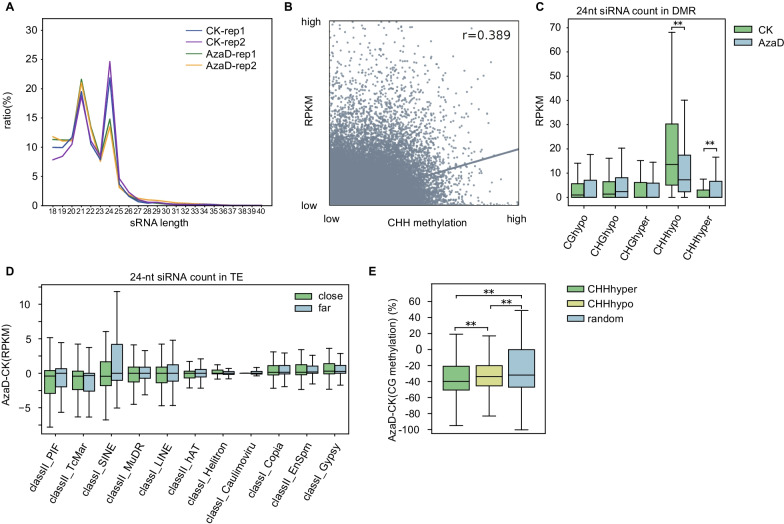
24-nt siRNAs were altered as a response to CG methylation loss. **a** siRNA profile of AzaD treated and CK rice. 21–22-nt siRNAs were up-regulated while 24-nt siRNA were down-regulated after AzaD treatment. **b** Correlation between CHH methylation level and 24-nt siRNA abundance in DMRs. **c** 24-nt siRNA RPKM in different DMRs. The 24-nt siRNA count was associated with CHH methylation. **d** 24-nt siRNA RPKM changes of close and far TEs from different TE families. The distribution pattern was highly similar to that of CHH methylation in TEs from different families. **e** CG methylation level changes in CHH hyper DMRs, CHH hypo DMRs, and random regions. *p < 0.05, **p < 0.01 by Student’s t-test

RdDM pathway, which mainly involves 24-nt siRNAs, is a vital pathway in establishing de novo methylation, especially CHH methylation (Matzke and Mosher 2014; Cuerda-Gil and Slotkin 2016, Wendte and Pikaard 2017). To explore the mechanism of CHH methylation changes, we performed small RNA sequencing (sRNA-seq) on rice leaf tissue of CK and AzaD treated rice plants. The abundance of 21–22-nt siRNAs were found to be slightly increased while that of 24-nt siRNAs was decreased (Fig. 4A). The overall changes in 24-nt siRNA abundance was in correspondence with CHH demethylation, with a correlation coefficient of 0.389 (Fig. 4B, Additional file 1: Fig. S13). Next, we calculated regional abundance of 24-nt siRNAs in DMRs of each sequence context (CG, CHG, and CHH) in CK and AzaD treated rice plants. The highest RPKM of siRNAs was found in CHH hypo DMRs before treatment and the abundance was significantly down-regulated after AzaD treatment. A significant increase was also found in the abundance of 24-nt siRNA in CHH hyper DMRs, while no obvious differences in 24-nt siRNA abundance were found in other DMRs (Fig. 4C). Previously, mutation of *OSMET1-2* caused concordant change in CHH methylation and 24-nt siRNA abundance that concordant changing regions (CCRs) are more than opposite changing regions (OCRs) (Hu et al. 2014). In our data, similar result was found that 24-nt siRNA abundance was down-regulated in most CHH hypo DMRs and 24-nt siRNA abundance was up-regulated in most CHH hyper DMRs (Additional file 1: Figure S14). These results demonstrate that changes in CHH were regulated by 24-nt siRNAs. Considering the correlation between 24-nt siRNA abundance and CHH methylation, we constructed a boxplot of 24-nt siRNA RPKM for far and close TEs of each family (Fig. 4D), and the distribution was highly consistent with that of CHH methylation levels described above (Fig. 3E). These finding further confirmed the close correlation between CHH methylation and 24-nt siRNA abundance in TE regions.

Next, we compared CG methylation changes in CHH hyper/hypo DMRs and random regions, and the results showed that CG methylation levels of CHH hyper DMRs were more significantly decreased than CHH hypo DMRs and random regions (Fig. 4E). This illustrated that CHH hyper methylation was mainly found distributed across regions with severe CG methylation loss. This result also explains that the rice plants likely induced de novo CHH methylation with 24-nt siRNAs through the RdDM pathway to compensate for the severe loss of CG methylation and to stabilize the whole genome after AzaD treatment.

### AzaD Treatment Causes Severe Phenotype Changes in Kitaake Rice Plants

**Fig. 5:**
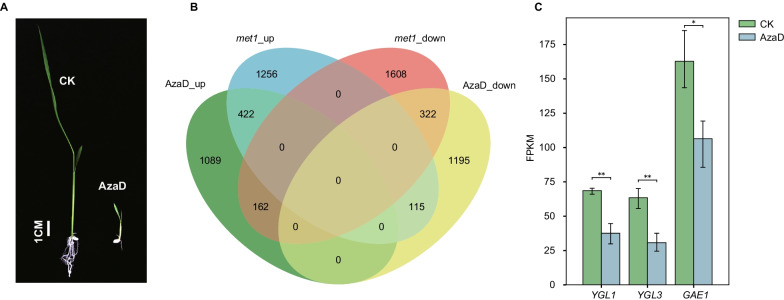
AzaD treatment causes severe phenotype changes in Kitaake. **a** Seedling phenotypes of CK and AzaD treated rice. Rice seedling and root growth was severely retarded after AzaD treatment, and the stem and leaves became etiolated. Scale bar = 1 cm. **b** Venn diagrams show the differentially expressed genes and their overlap between AzaD treated and *osmet1-2* mutant rice. **c** Expression levels of *OsYGL1*, *OsYGL3*, and *OsGAE1* genes. *p < 0.05, **p < 0.01 by Student’s *t*-test

Rice seedling and root growth was severely retarded after AzaD treatment, and the stem and leaves became etiolated (Fig. 5A). To characterize the effects of AzaD-induced methylome changes on gene expression, we performed RNA-sequencing (RNA-seq) on leaf tissues of CK and AzaD treated rice plants. Differentially expressed genes (DEGs) were identified in AzaD treated plants (Fig. 5B). In all, 1876 genes showed significantly up-regulated expression levels and 1,730 genes were down-regulated. We further compared the overlap rates of DEGs in AzaD treated plants and *osmet1-2* mutants. In total, 1876 genes were up-regulated in the AzaD treatment and 422 genes were up-regulated in *osmet1-2* mutants; only a few genes (162) showed opposite regulation patterns and 1,089 genes showed no obvious change in *osmet1-2* mutants (Fig. 5B). In all, 1730 genes were down-regulated after AzaD treatment, of which 322 genes were also down-regulated in *osmet1-2* mutants; 115 genes were up-regulated in *osmet1-2* mutants and the remaining 1,195 genes showed no change (Fig. 5B). GO analysis showed that genes up-regulated in both backgrounds were involved in ‘secondary metabolic biosynthetic process’, ‘secondary metabolite process’, ‘organic acid biosynthetic process’, ‘carboxylic acid biosynthetic process’, among others (Additional file 1: Figure S15A). Genes down-regulated in both backgrounds were involved in ‘regulation of hormone levels’, ‘hormone metabolic process’, and ‘meta ion homeostasis’, among others (Additional file 1: Figure S15B). Genes only up-regulated in AzaD treated plants were involved in ‘response to water deprivation’, ‘response to water’, and ‘response to heat’, among others, suggesting that AzaD treatment activated stress response genes (Additional file 1: Figure S16A). Genes only up-regulated in *osmet1-2* mutants were involved in ‘cell cycle process’, ‘chromosome’, ‘cytoskeleton’, and ‘DNA replication’, among others, which are all essential processes and components for survival (Additional file 1: Figure S16A). However, genes involved in biological processes such as ‘response to drug’, ‘response to water’, ‘response to lipid’, and ‘response to cold’ were down-regulated in *osmet1-2* mutants (Additional file 1: Figure S17B). Genes involved in ‘thylakoid’ and ‘envelope’ were down-regulated in AzaD treated plants (Additional file 1: Figure S16B). Genes related to abiotic stress were mainly up-regulated after AzaD treatment but were down-regulated in *osmet1-2* mutants. These different gene regulation patterns suggest there are different impacts of AzaD treatment and mutation of *OsMET1-2* on the whole genome.

According to RNA-seq analysis, yellow-green leaf 1 (*YGL1*) and *YGL3* were found to be down-regulated after treatment, as was the gibberellin-enhanced gene *OsGAE1* (Fig. 5C). Interestingly, the repressed expressions of these genes were not caused by methylation changes, since methylation was down-regulated rather than up-regulated, which should have caused up-regulated gene expression (Additional file 1: Figure S18). These down-regulated genes shared a common characteristic in that a transposon was demethylated in the body flanking the gene, indicating that the transposons might disturb the expression of their flanking gene and cause corresponding phenotypes.

## Discussion

To investigate whether AzaD treatment could mimic *osmet1-2* mutation, we comprehensively compared the similarities and differences in methylation changes between AzaD treatment and *osmet1-2* plants. The results showed that there was a high overlap ratio of hypo DMRs, especially CG hypo DMRs, indicating consistency in the regions of methylation changes affected by AzaD treatment and *osmet1-2* mutation. In addition, there was a high rate of overlap between the differentially expressed genes of AzaD treated plants and *osmet1-2* mutants, which regulate metabolic processes and hormone levels. These results illustrate the feasibility of AzaD treatment mimicking the *osmet1-2* mutation, and being used as a tool to avoid the difficulty of obtaining *met1-2* homozygous mutations in rice (Li et al. 2014; Hu et al. 2014).

In addition to the similarities, there were also key differences between AzaD treatment and *osmet1-2* mutation. Compared with *osmet1-2* mutation, the down-regulation of CG methylation by AzaD treatment was more moderate, which might be the reason why AzaD treated plants could survive more easily. Additionally, AzaD treatment showed a significant effect on CHH upregulation in class I TEs, which was not observed in *osmet1-2* mutants (Additional file 1: Figure S6). This difference was probably due to plants treated with AzaD responding to it as a stressor and activating pathways involving CHH methylation that differed from the response in *osmet1-2* mutants. The reason why CHH hyper methylation primarily happens in class I TEs may be that class I TEs may cause more severe perturbations to the genome. Plants establish de novo CHH methylation in retrotransposon bodies through the RdDM pathway as a response to compensate for severe methylation loss and to stabilize the genome, although this requires further research. Moreover, genes involved in stress responses were up-regulated in AzaD treated plants, suggesting AzaD is perceived as a stressor. Conversely, stress response genes were down-regulated in *osmet1-2* mutants, while basic functions such as ‘cell cycle process’ and ‘DNA replication’ were up-regulated. The different gene regulation patterns in *osmet1-2* mutants indicate that mutation of *OsMET1-2* can cause severe repression of rice development causing the genome to activate these basic processes to survive.

Another similar chemical inhibitor, 5-AZA, was used in *Arabidopsis* plants and triggered genome wide demethylation (Griffin et al. 2016). The CG methylation loss was universal across the whole genome, but CHH loss was more severe in the pericentromeric region, which differs from our results. A possible explanation for this is that there is a difference between dicot and monocot plant genomes and methylation inhibitors that cause activation of various genes in rice and *Arabidopsis* plants. This study provides a detailed look into the application of AzaD for research purposes with regard to demethylation, TE activation, and CHH methylation pathways.

## Conclusion

AzaD was used to treat Kitaake rice and WGBS was performed to study the global demethylation happened in Kitaake genome after treatment. We compared the AzaD treated rice plants with *osmet1-2* mutants, illustrating that there are similar CG hypomethylation and distribution throughout the whole genome. Additionally, Class I retrotransposons that mainly distribute in heterochromotain regions and far away from genes were activated more than class II TEs. Meanwhile, CHH methylation was elevated in class I retrotransposons and regions with severe CG loss, trying to compensate for the impact caused by global demethylation.

## Materials and Methods

### Plant Material and AzaD Treatment

Healthy dry seeds of rice cultivar Kitaake (*O. sativa* ssp. *geng/japonica*) were dehulled and sterilized by submerging in 70% ethanol for 1 min, followed by 10% sodium hypochlorite for 60 min. The seeds were then washed in distilled water three times (1 min per wash). The seeds were incubated on half-strength MS medium with or without 40 mg/L AzaD in a growth chamber kept at a consistent 25 °C under a 12:12 h light/dark cycle.

### BS-seq and Methylation Data Analysis

Genomic DNA (gDNA) was extracted from AzaD treated and non-treated seedlings 7 days post incubation using the CTAB method. An EZ DNA Methylation-Gold Kit (ZYMO) was used for DNA sodium bisulfite treatment, according to the manufacturer’s instructions. Samples were submitted to Novogene Co. Ltd. (Tianjin) for bisulfate sequencing using the Illumina Nova 6000 platform.

Low quality data was removed from the raw read data, and the clean read data was aligned to the reference genome of Kitaake rice (Kitaake version 2.0) (Li et al. 2017) using Bismark version 0.17.0 (Krueger and Andrews 2011) using standard settings. Methylation information was extracted from BAM files and CGmap was generated from coverage files by Bismark. Further, differentially methylated region (DMR) identification was carried out using R package dmrfinder (Gaspar and Hart 2017). DMR was calculated when the differences in methylation level reached 40% for CG, 20% for CHG, and 10% for CHH, respectively. DMRs were merged together if their distance was less than 50 bp. Genome-wide methylation levels were calculated using weighted methylation (Schultz et al. 2012).

### TE Annotation and Analysis

Synteny analysis between the Kitaake and Nipponbare genomes (Kawahara et al. 2013) was constructed using SYMAP (Soderlund et al. 2011) and TEs were selected according to the syntenic results. Annotated repeat sequences in Kitaake were aligned to the repeatmasker TE protein database and merged together. Transcript were predicted using GMAP (Wu and Watanabe 2005) with Kitaake RNA-seq data, and compared. The annotated.gtf file was used to select transcripts with no overlap with genes as TEs. Repeatmasker (Smit et al. 1996) was used to annotate the transcripts with the highest Z-Score. The results were merged together and 19,788 TEs in total were identified.

TE count from different superfamilies were calculated and listed in Additional file 1: Table S1 and Additional file 1: Table S2. All annotated TEs were divided into far/close groupings according to their distance to the closest gene, while TEs expressed in at least one situation were calculated as activated/not activated, and TEs not expressed in both the AzaD treatment and CK were considered ‘dead’ TEs and removed.

### RNA-seq and Data Analysis

Total RNA was extracted from AzaD treated and non-treated seedlings 7 d after incubation using the Plant RNA Kit (Omega) and treated with an RNA-free DNase set (Omega) to remove DNA contamination according to the manufacturer’s protocol. The samples were submitted to Novogene Co. Ltd. (Tianjin) for library preparation and sequencing using the Illumina Nova 6000 platform.

RNA-seq reads were aligned to the reference genome using Hisat2 (version 2.2.0) (Kim et al. 2015) using default parameters. BAM files were processed with Cufflinks (version 2.2.1) (Trapnell et al. 2012) to perform differential expression analysis. R packages clusterProfiler (Wu et al. 2021) and enrichplot were used for GO analysis.

### RNA Extraction and RT-qPCR

Total RNA was extracted from AzaD treated and non-treated seedlings 7 d after incubation using the Plant RNA Kit (Omega) according to the manufacturer's instructions and was reverse transcripted using reverse transcription kit (TaKaRa). Gene expression levels were analysed using SYBR Green PCR Kit (Bio-RAD). The Actin gene was used as the internal reference to normalize gene expression data. The expression levels of genes were compared using ΔΔCt method. PCR primer sets for gene amplification are given in Additional file 1: Table S3.

### sRNA-seq and Data Analysis

sRNA fastq files were aligned to the reference genome using bowtie (version 2.4.4) (Langmead and Salzberg 2012) with parameters ‘-n 0 -m 1 –best –strata’. Duplicates were removed using Samtools (Li et al. 2009), and 21–22 and 24-nt siRNAs were separated according to their lengths. The siRNA normalized count in specific regions was calculated using Bedtools (version 2.29.2) (Quinlan and Hall 2010) and custom python scripts.

## Supplementary Information

**Additional file 1**. **Fig. S1.** Fold changes of CG, CHG, and CHH methylation levels on every chromosome of Kitaake after AzaD treatment. **Fig. S2.** Fold changes of CG, CHG, and CHH methylation levels on every chromosome of Nipponbare after mutation of *OsMET1-2*. **Fig. S3.** DMR distribution in different parts of the genomes of AzaD treated and *osmet1-2* mutant rice plants. **Fig. S4.** DMR overlap ratio between AzaD treated and *osmet1-2* mutant rice according to collinear comparisons between genomes of Kitaake and Nipponbare rice cultivars. **Fig. S5.** Gene expression levels in CK, AzaD treated, WT, and *osmet1-2* mutant rice plants. **Fig. S6.** CG, CHG, and CHH methylation levels of class I retrotransposons and class II DNA transposons in *osmet1-2* mutant rice plants. **Fig. S7.** Distribution of class I and class II TEs on every chromosome of Kitaake. **Fig. S8.** The methylation change in activated and not activated TEs. **Fig. S9.** Average distances of TEs from their closest genes. **Fig. S10.** Methylation level of TEs from different superfamilies in Kitaake. **Fig. S11.** CG methylation level changes of TEs. **Fig. S12.** CHH methylation level changes of TEs in *osmet1-2* mutant rice plants. **Fig. S13.** Correlation between 24-nt siRNA RPKM and methylation levels in DMRs. **Fig. S14.** The siRNA count and CHH methylation level change in CHH DMRs in AzaD treated plants. **Fig. S15.** GO enrichment analysis of DEGs in both AzaD treated and *osmet1-2* mutant rice plants. **Fig. S16.** GO enrichment analysis of DEGs in AzaD treated rice plants. **Fig. S17.** GO enrichment analysis of DEGs in *osmet1-2* mutant rice plants. **Fig. S18.** Examples of DNA methylation profiles in three down-regulated genes. **Table S1.**. TE count from different superfamilies in CK and AzaD treatment plants. **Table S2.**. TE count from different superfamilies in WT and *osmet1-2* mutant plants. **Table S3.**. PCR primer sets used in RT-qPCR.

## Data Availability

The datasets supporting the conclusions of this article are available in the National Center for Biotechnology information (NCBI) database (https://www.ncbi.nlm.nih.gov/) under Sequence Read Archive (SRA) BioProject “PRJNA781032” and Beijing Institute of Genomics Data Center (http://bigd.big.ac.cn) under BioProject “PRJCA007230”.

## Abbreviations

RdDM: RNA-directed DNA methylation
AzaD: 5-Aza-2′-deoxycytidine
TE: Transposable element
5-AZA: 5-Azacytidine
ZEB: Zebularine
DMR: Differentially methylated region
WGBS: Whole-genome bisulfite sequencing
DEG: Differentially expressed gene
