# Supplementary material for: The Methylation Inhibitor 5-Aza-2′-Deoxycytidine Induces Genome-Wide Hypomethylation in Rice
Source: Rice (N Y). 2022 Jul 2;15:35. doi: 10.1186/s12284-022-00580-6 (PMC9250569; doi:10.1186/s12284-022-00580-6)
Supplement: Supplementary file 1 — Additional file 1. Fig. S1. Fold changes of CG, CHG, and CHH methylation levels on every chromosome of Kitaake after AzaD treatment. Fig. S2. Fold changes of CG, CHG, and CHH methylation levels on every chromosome of Nipponbare after mutation of OsMET1-2. Fig. S3. DMR distribution in different parts of the genomes of AzaD treated and osmet1-2 mutant rice plants. Fig. S4. DMR overlap ratio between AzaD treated and osmet1-2 mutant rice according to collinear comparisons between genomes of Kitaake and Nipponbare rice cultivars. Fig. S5. Gene expression levels in CK, AzaD treated, WT, and osmet1-2 mutant rice plants. Fig. S6. CG, CHG, and CHH methylation levels of class I retrotransposons and class II DNA transposons in osmet1-2 mutant rice plants. Fig. S7. Distribution of class I and class II TEs on every chromosome of Kitaake. Fig. S8. The methylation change in activated and not activated TEs. Fig. S9. Average distances of TEs from their closest genes. Fig. S10. Methylation level of TEs from different superfamilies in Kitaake. Fig. S11. CG methylation level changes of TEs. Fig. S12. CHH methylation level changes of TEs in osmet1-2 mutant rice plants. Fig. S13. Correlation between 24-nt siRNA RPKM and methylation levels in DMRs. Fig. S14. The siRNA count and CHH methylation level change in CHH DMRs in AzaD treated plants. Fig. S15. GO enrichment analysis of DEGs in both AzaD treated and osmet1-2 mutant rice plants. Fig. S16. GO enrichment analysis of DEGs in AzaD treated rice plants. Fig. S17. GO enrichment analysis of DEGs in osmet1-2 mutant rice plants. Fig. S18. Examples of DNA methylation profiles in three down-regulated genes. Table S1.. TE count from different superfamilies in CK and AzaD treatment plants. Table S2.. TE count from different superfamilies in WT and osmet1-2 mutant plants. Table S3.. PCR primer sets used in RT-qPCR. [file 12284_2022_580_MOESM1_ESM.pdf]

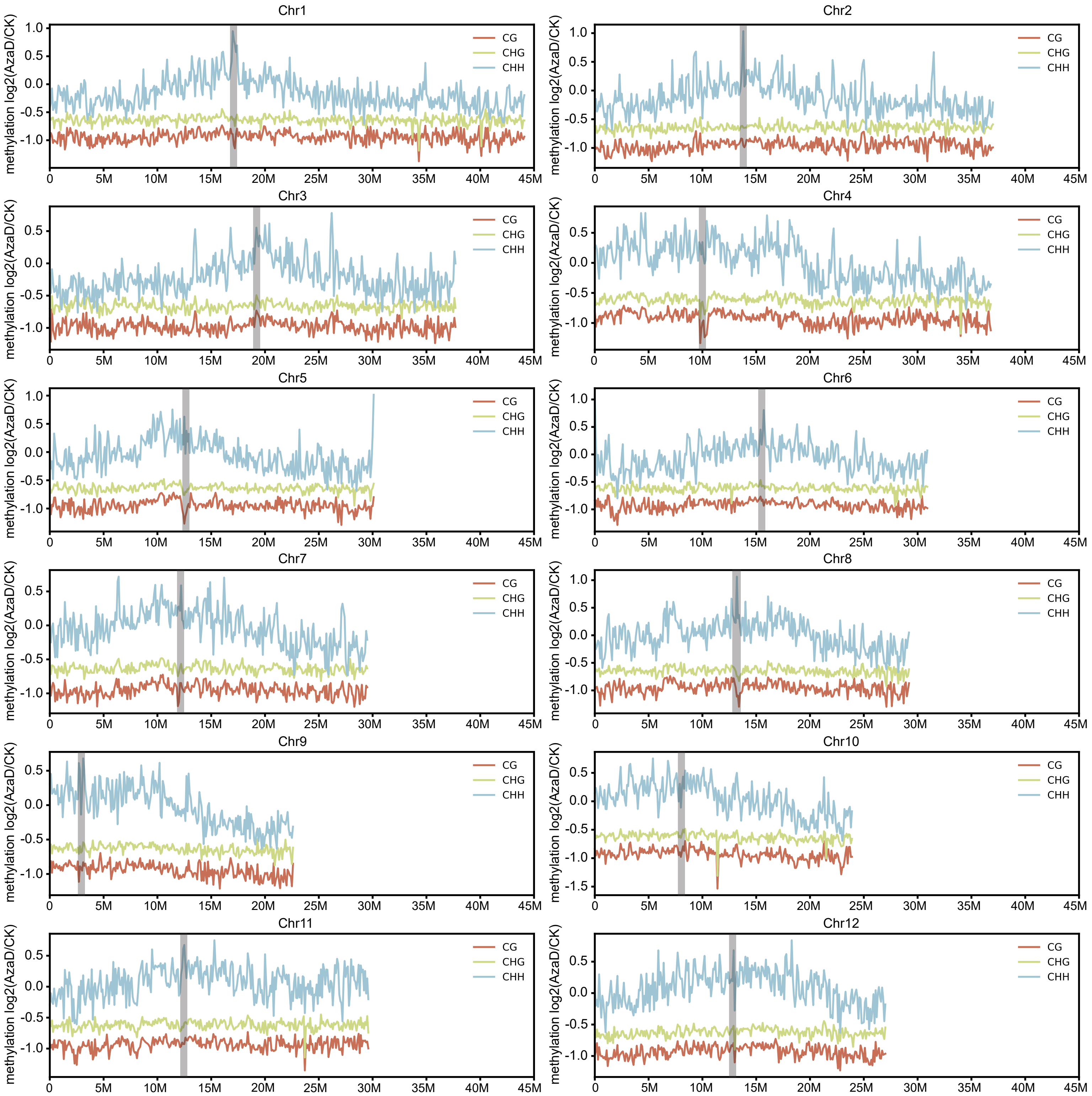

Figure S1. Fold changes of CG, CHG, and CHH methylation levels on every chromosome of Kitaake after AzaD treatment

The genome wide changes of DNA methylation levels in AzaD treated rice and CK are shown with line charts. The grey boxes mark the centromere regions.

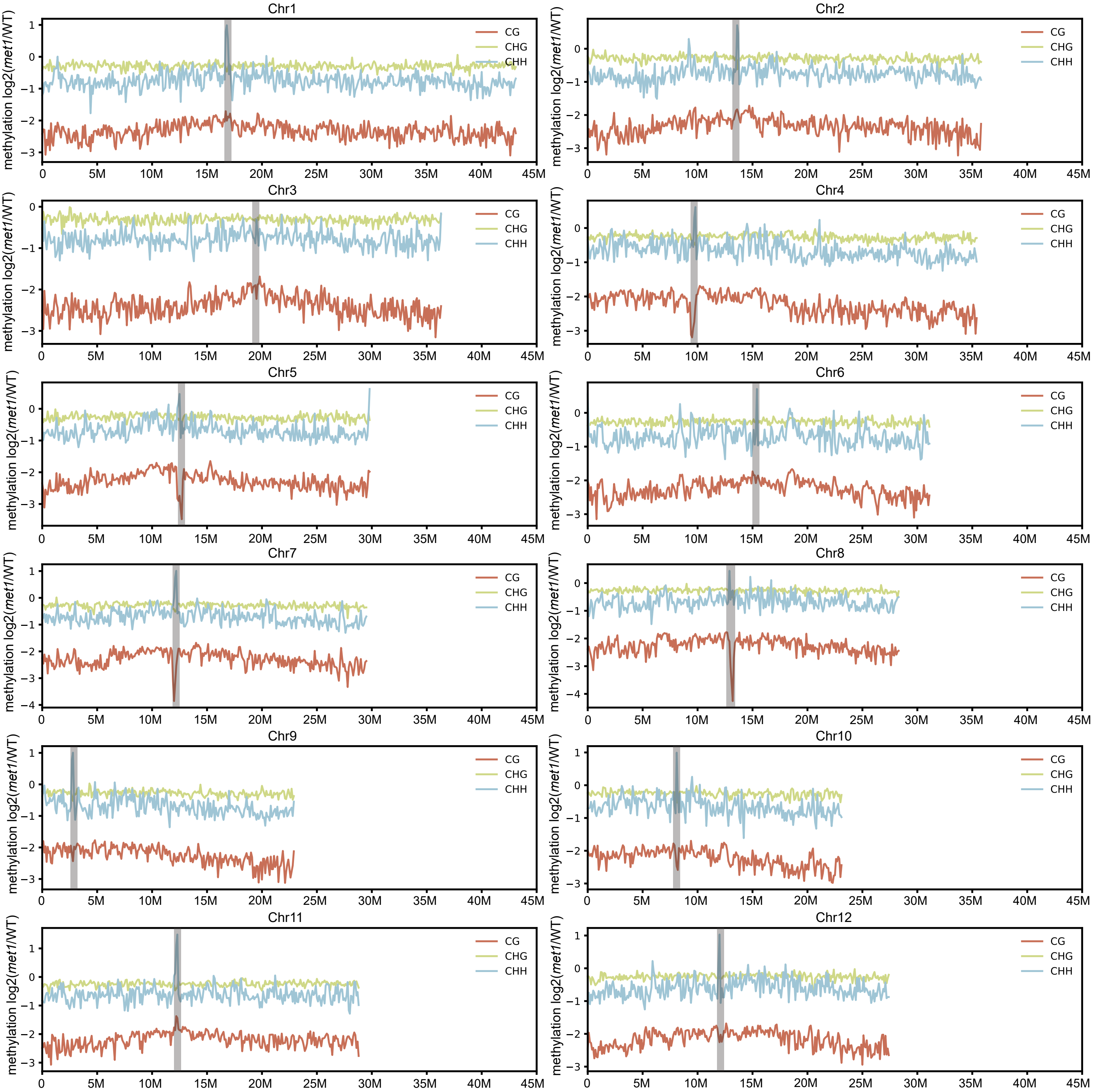

Figure S2. Fold changes of CG, CHG, and CHH methylation levels on every chromosome of Nipponbare after mutation of *OsMET1-2*

The genome wide changes of DNA methylation levels in *osmet1-2* mutant rice and WT are shown with line charts. The grey boxes mark the centromere regions.

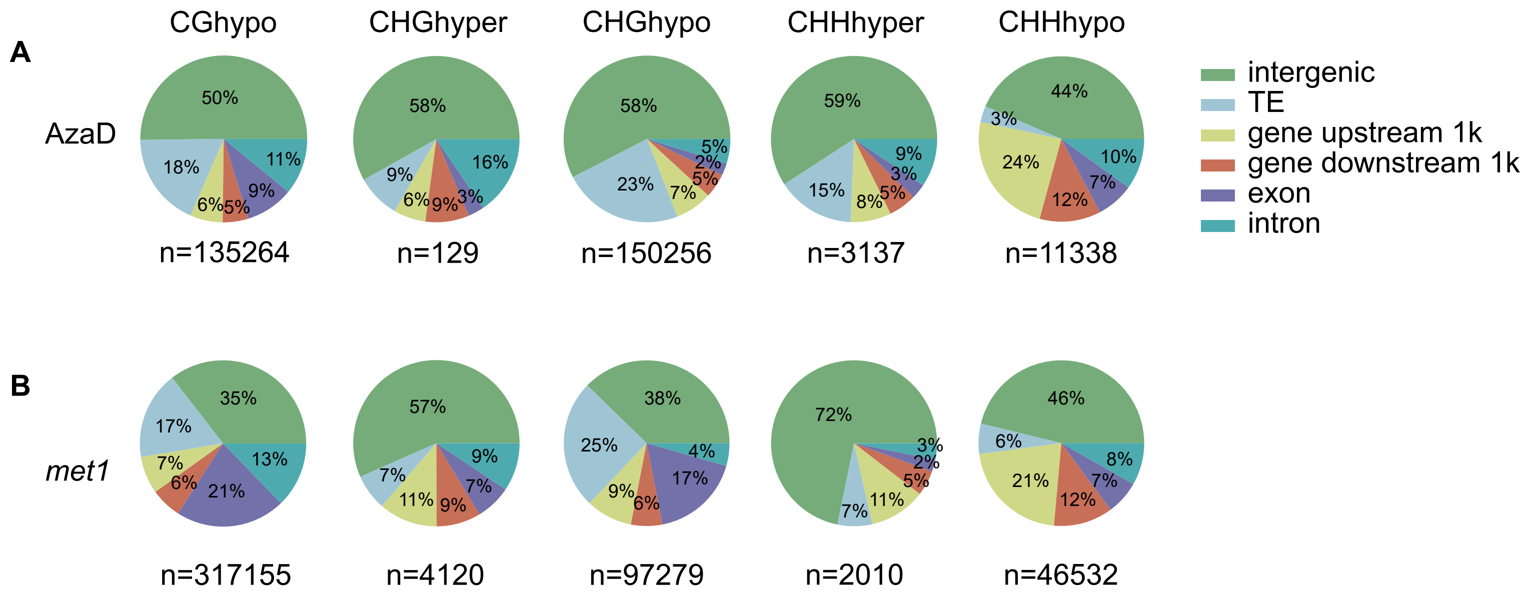

Figure S3. DMR distribution in different parts of the genomes of AzaD treated and *osmet1-2* mutant rice plants

Pie charts show the distribution of identified DMRs in different parts of the genomes in **(A)** AzaD treated rice and **(B)** *osmet1-2* mutant rice plants.

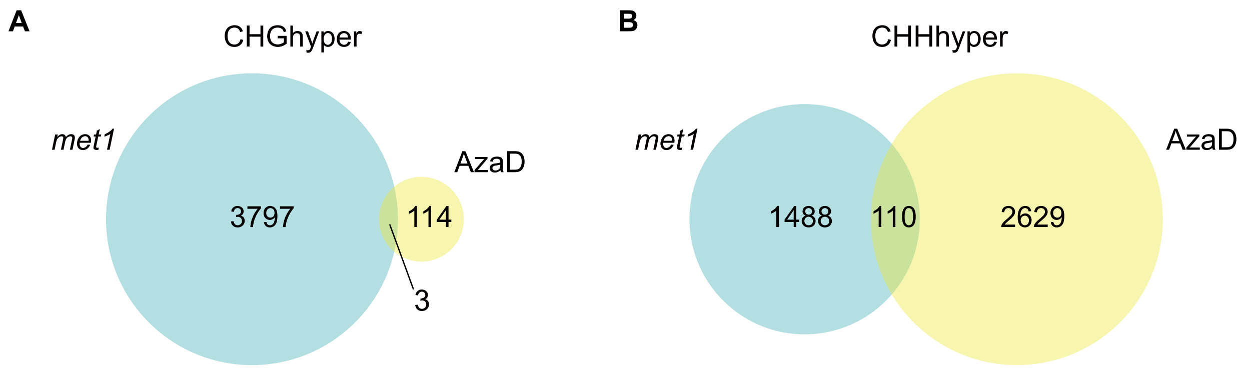

Figure S4. DMR overlap ratio between AzaD treated and *osmet1-2* mutant rice according to collinear comparisons between genomes of Kitaake and Nipponbare rice cultivars

Venn diagrams show the overlap ratio of **(A)** CHG hyper DMRs and **(B)** CHH hyper DMRs between AzaD treated and *osmet1-2* mutant rice.

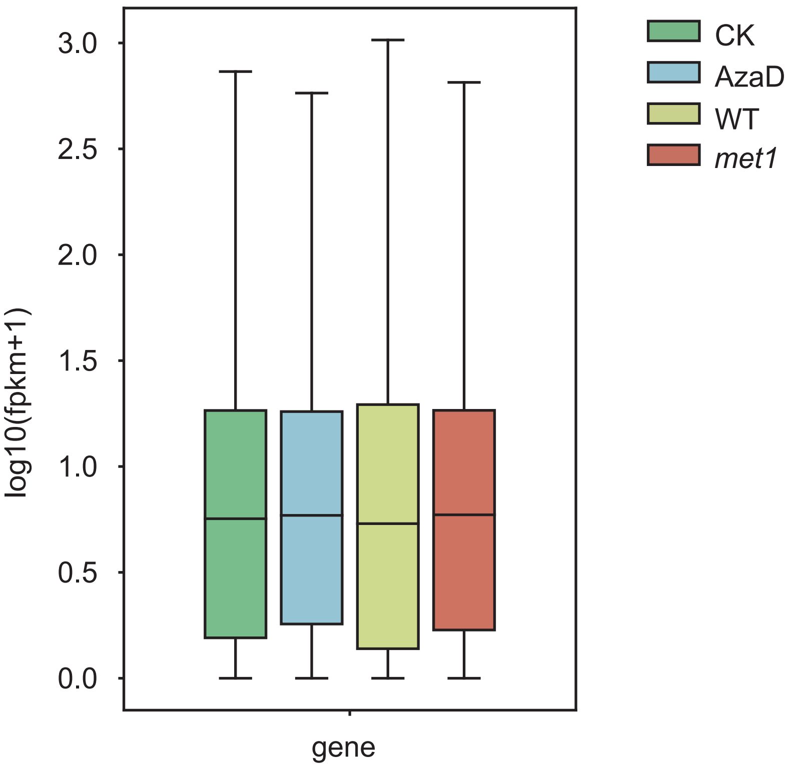

Figure S5. Gene expression levels in CK, AzaD treated, WT, and *osmet1-2* mutant rice plants
Boxplot shows the global gene expression levels in CK, AzaD treated, WT, and *osmet1-2* mutant rice plants.

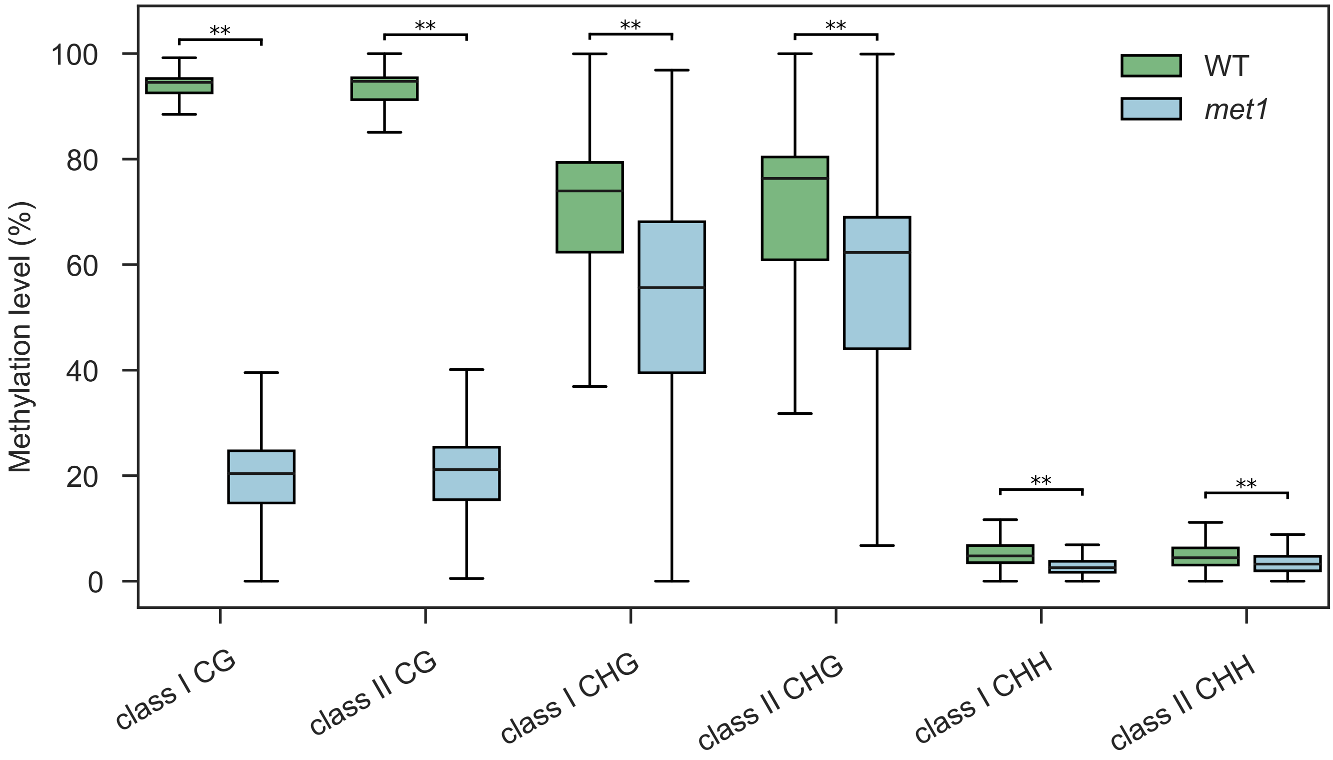

Figure S6. CG, CHG, and CHH methylation levels of class I retrotransposons and class II DNA transposons in *osmet1-2* mutant rice plants

CG, CHG, and CHH methylation levels of class I retrotransposons and class II DNA transposons in WT and *osmet1-2* mutant rice plants. *p<0.05, **p<0.01 by Student's t-test.

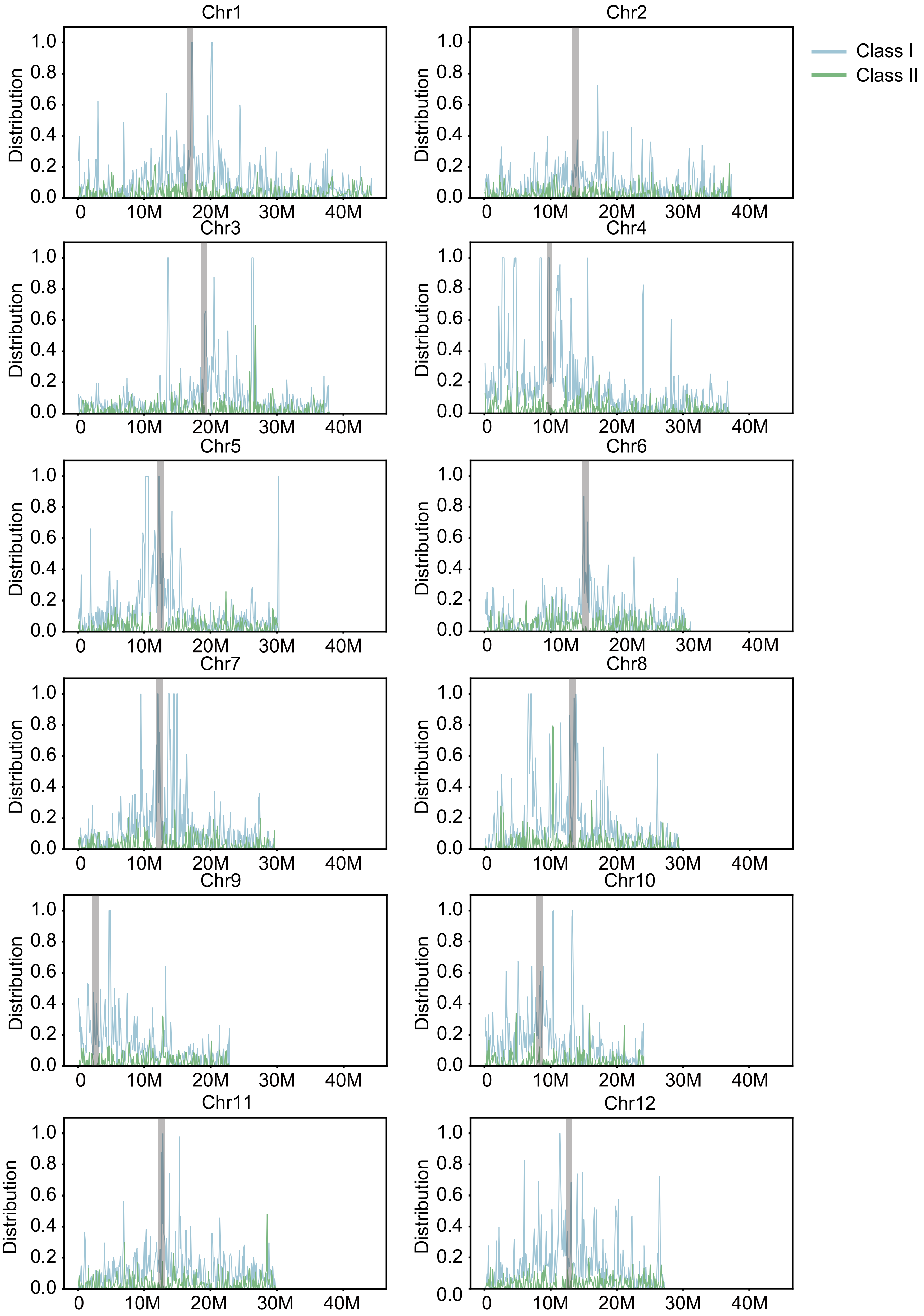

Figure S7. Distribution of class I and class II TEs on every chromosome of Kitaake

Line charts show the distribution of class I and class II TEs on each chromosome of Kitaake. Chromosomes were divided into 100 kb windows. The y axis shows the total length of TEs in each 100 kb window. The grey boxes mark the centromere regions.

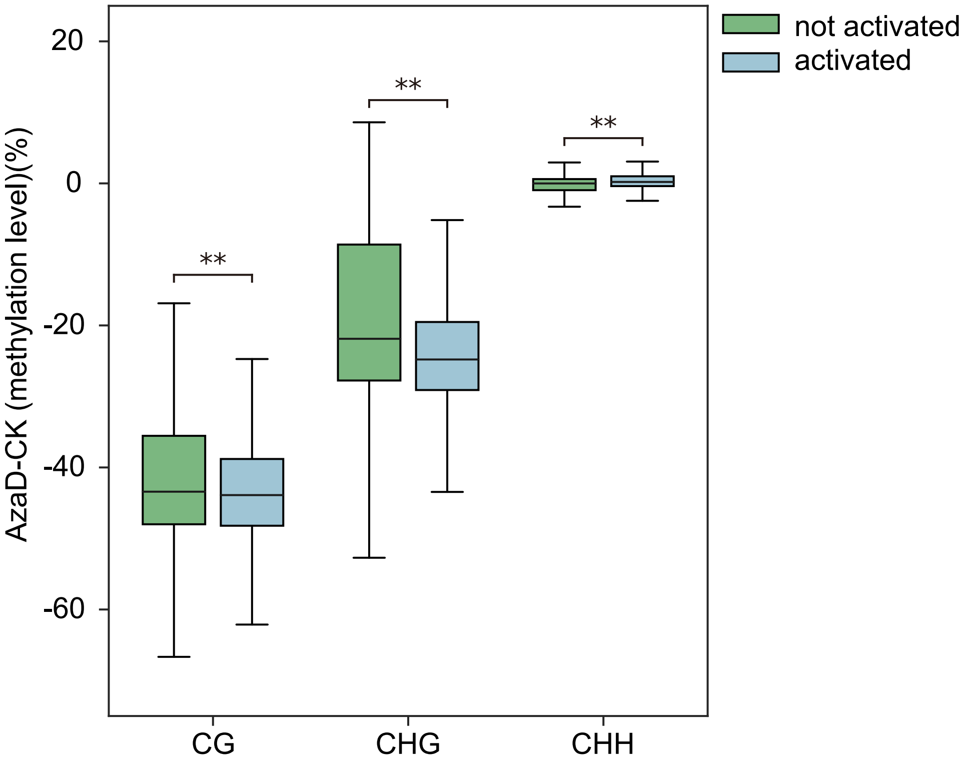

Figure S8. The methylation change in activated and not activated TEs
The CG and CHG methylation was significantly down-regulated more in activated TEs than not activated TEs. The CHH methylation in not activated TEs showed more reduction than in activated TEs. *p<0.05, **p<0.01 by Student's t-test.

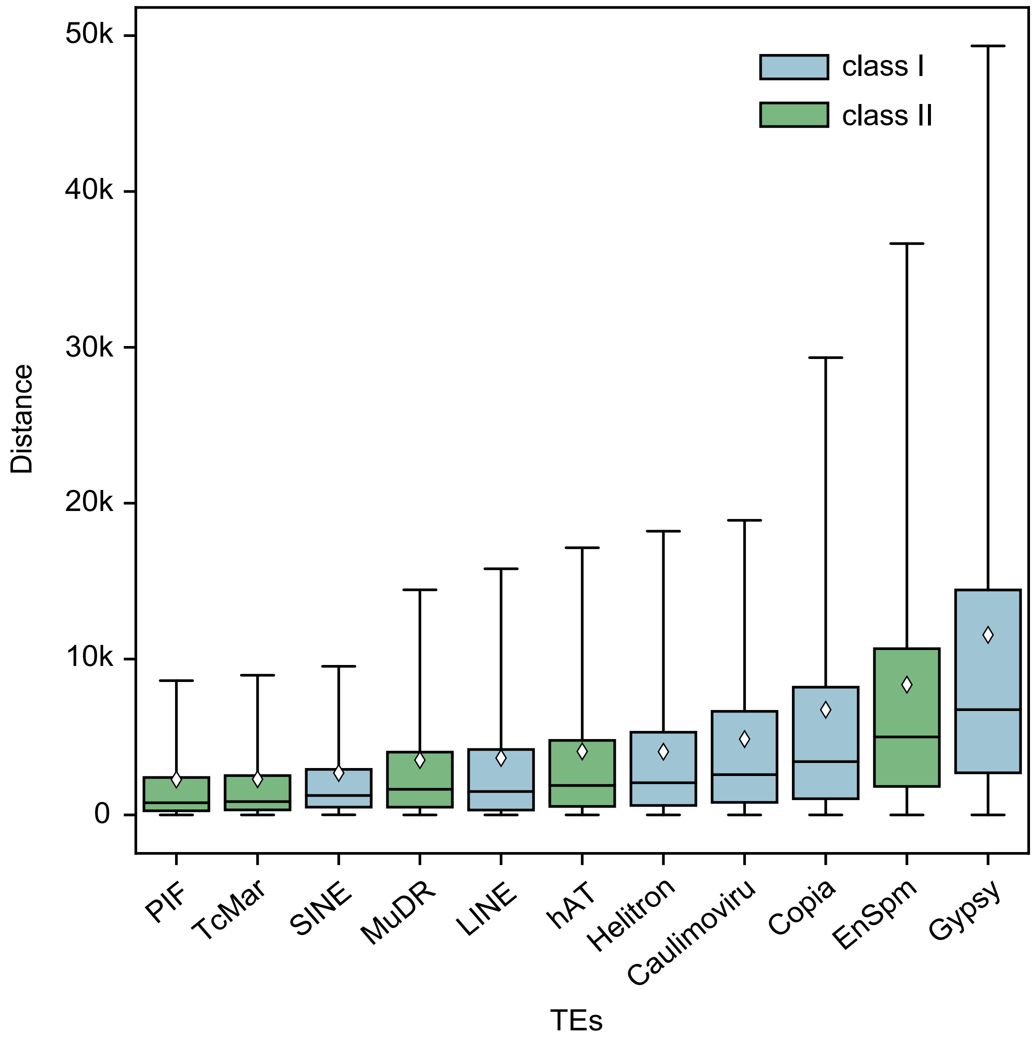

Figure S9. Average distances of TEs from their closest genes

Boxplot shows the distances of class I and class II TEs from their closest genes. The diamonds show the average distances. TE families were ordered according to the distances from nearest genes.

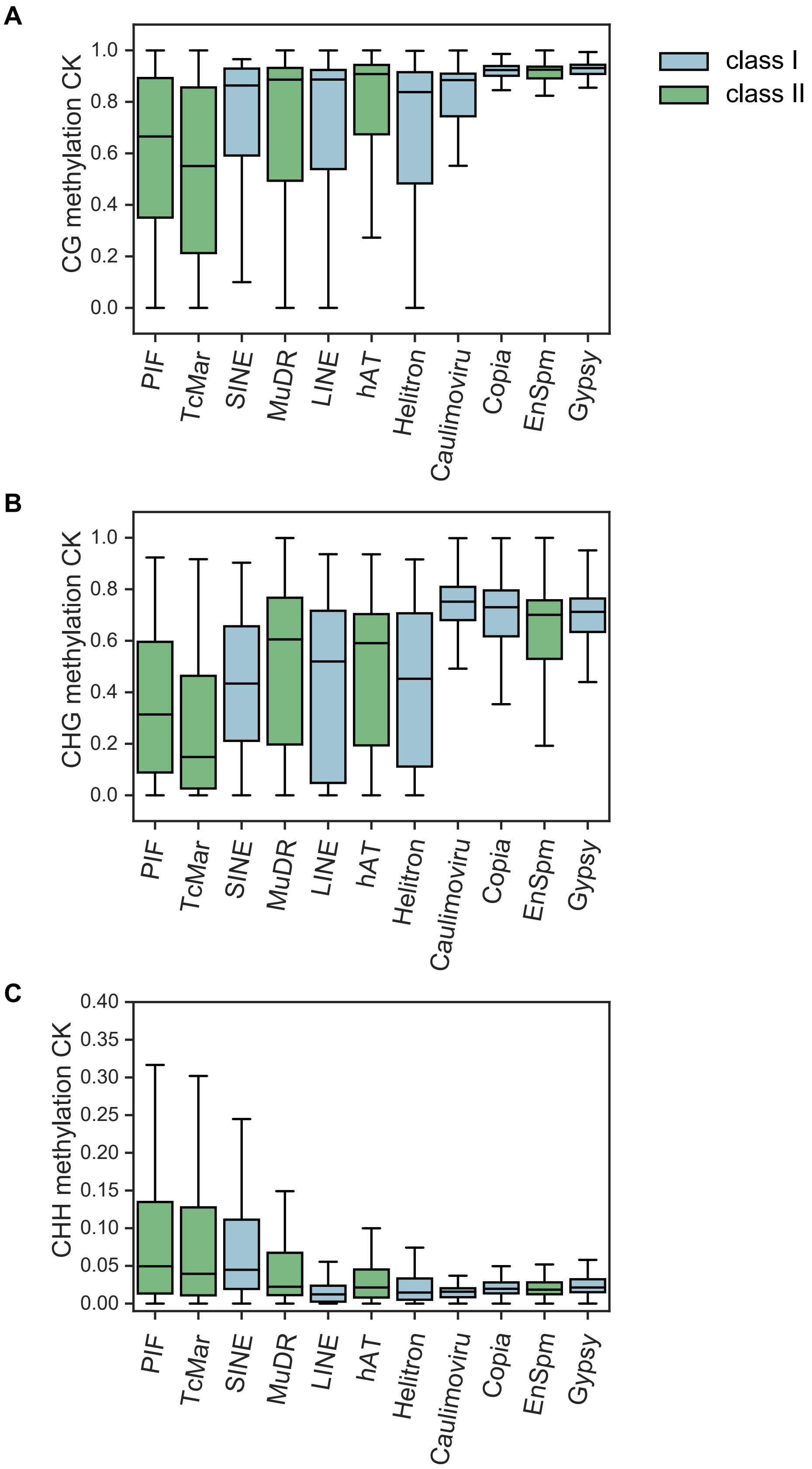

Figure S10. Methylation level of TEs from different superfamilies in Kitaake
(A) CG methylation, **(B)** CHG methylation, and **(C)** CHH methylation levels of TEs from different superfamilies in Kitaake. The x axis is ordered according to the average distance of TE superfamilies to the closest gene.

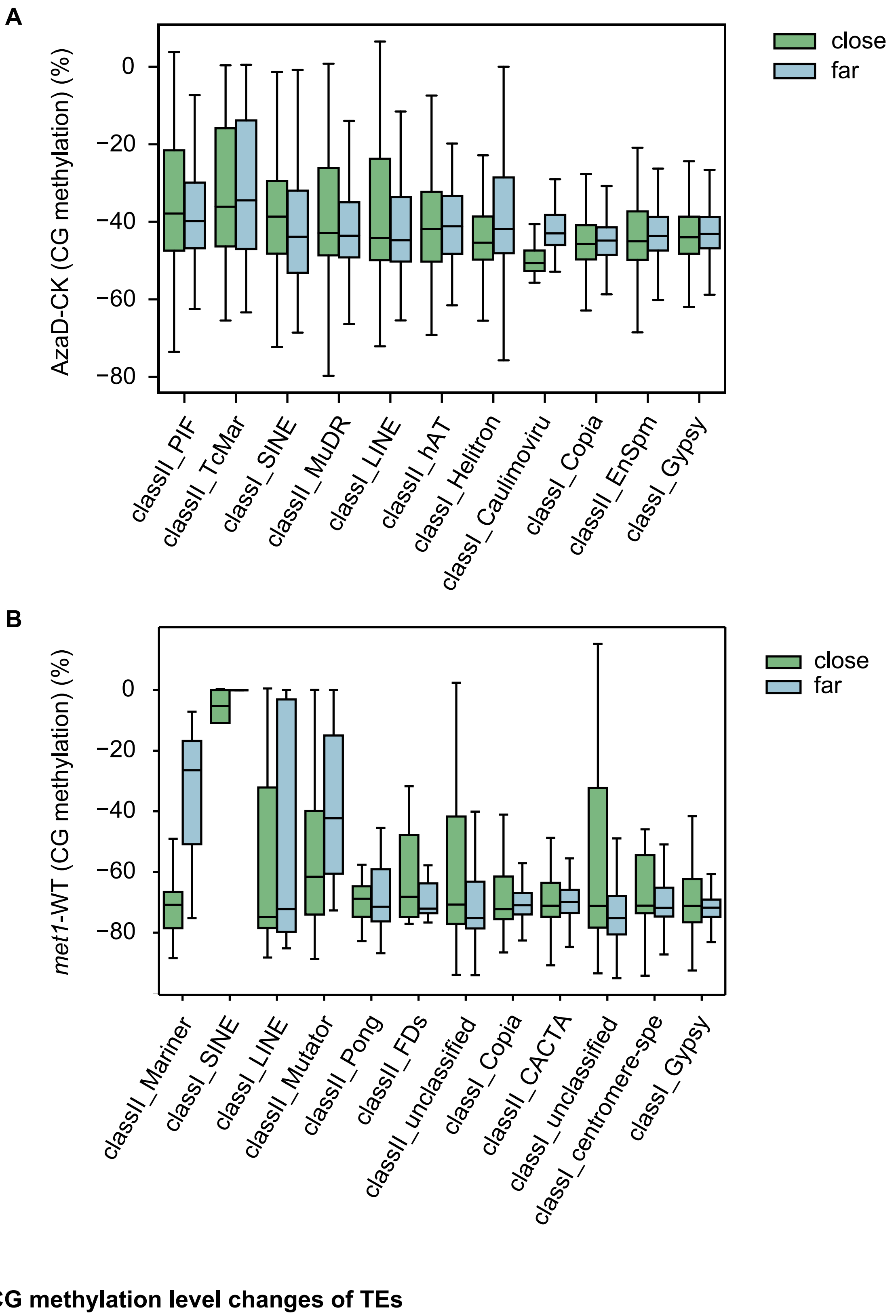

Figure S11. CG methylation level changes of TEs

CG methylation level changes of close and far TEs from each superfamily in **(A)** AzaD treated and **(B)** *osmet1-2* mutant rice plants. The x axis is ordered according to the average distance of TE superfamilies to the closest gene.

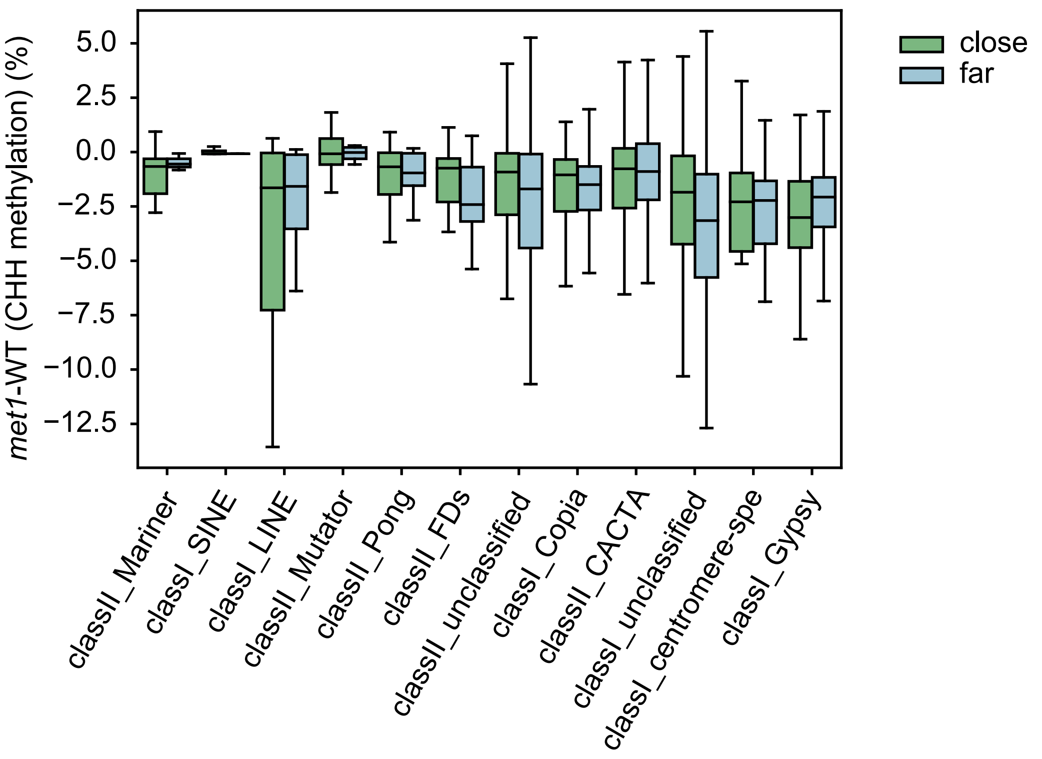

Figure S12. CHH methylation level changes of TEs in *osmet1-2* mutant rice plants
CHH methylation level changes of close and far TEs from different families in *osmet1-2* mutant rice plants. The x axis is ordered according to the average distance of TE superfamilies to the closest gene.

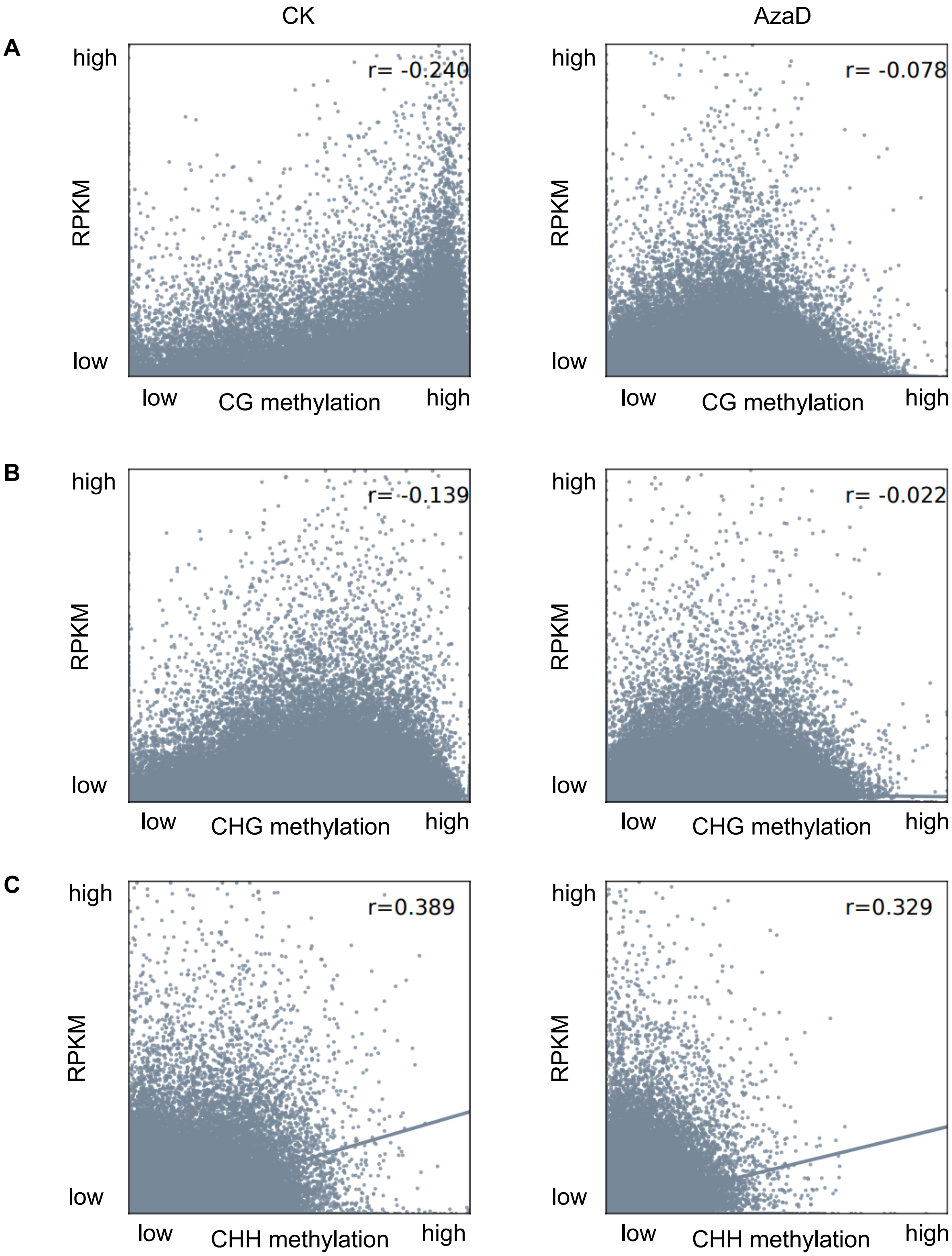

Figure S13. Correlation between 24-nt siRNA RPKM and methylation levels in DMRs

Correlation between 24-nt siRNA RPKM and **(A)** CG methylation, **(B)** CHG methylation, and **(C)** CHH methylation levels in all DMRs.

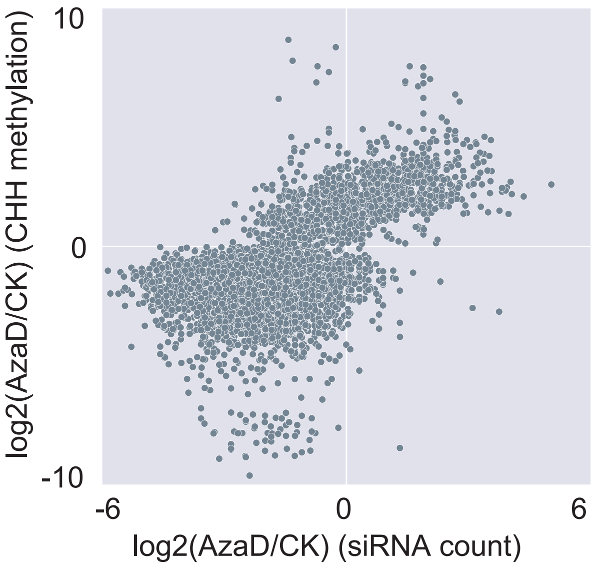

Figure S14. The siRNA count and CHH methylation level change in CHH DMRs in AzaD treated plants
The siRNA count and CHH methylation level change in CHH DMRs. The x axis represents the change of siRNA count. The y axis represents the change of CHH methylation.

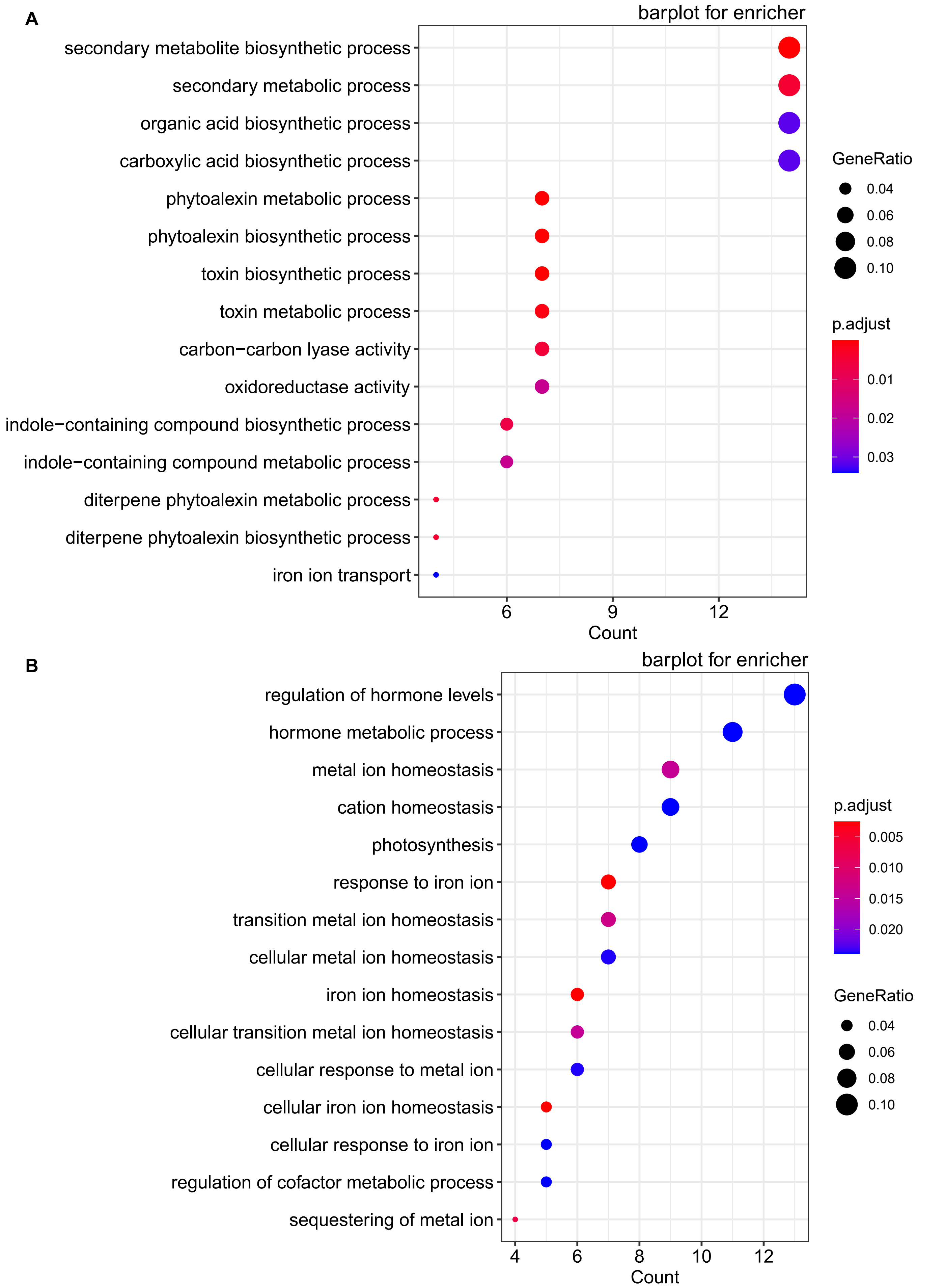

Figure S15. GO enrichment analysis of DEGs in both AzaD treated and *osmet1-2* mutant rice plants
Bubble plot shows the GO enrichment results of **(A)** up-regulated and **(B)** down-regulated genes in both AzaD treated and *osmet1-2* mutant rice plants. The x axis represents the number of genes in the specific GO item. The y axis represents all the enriched GO items.

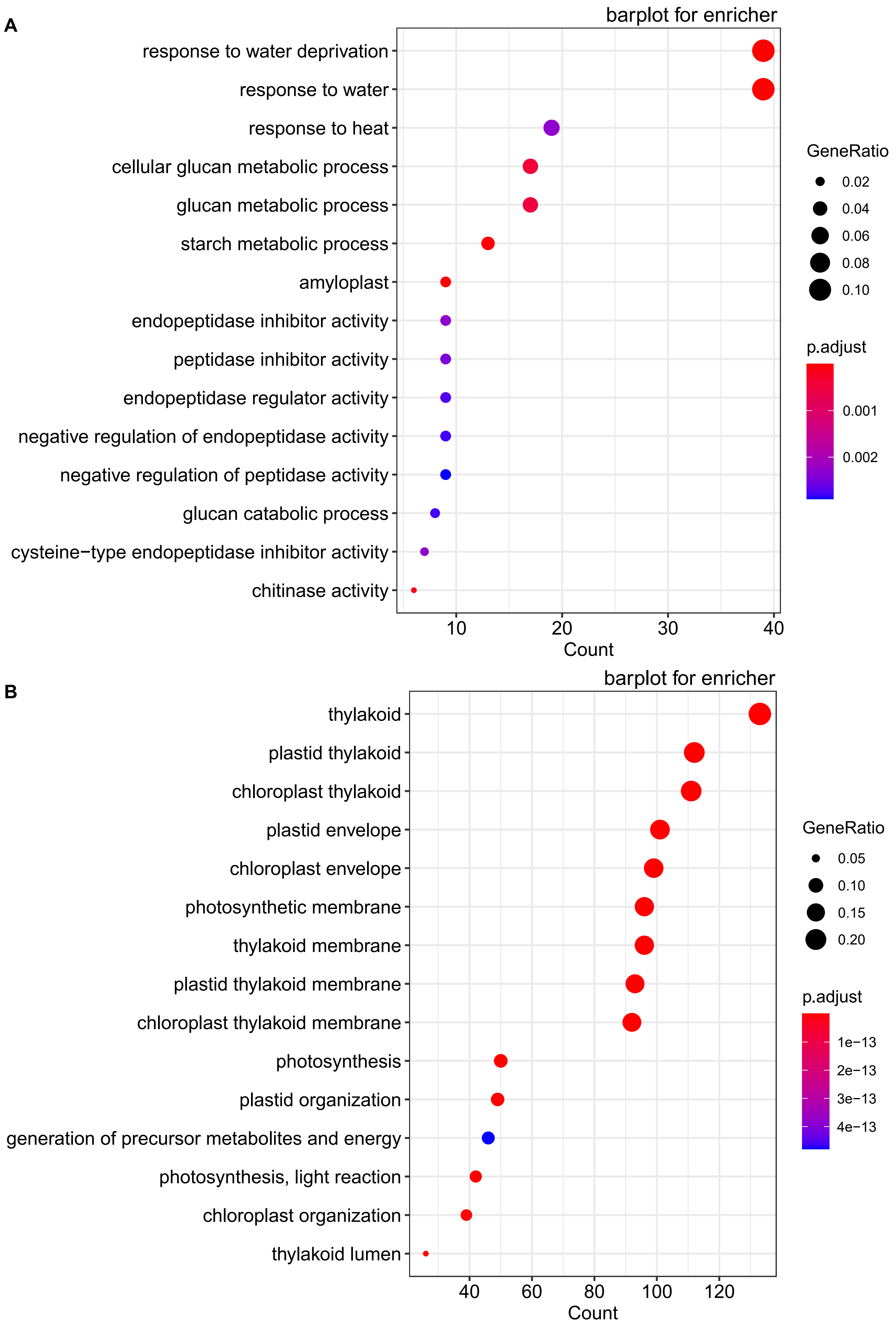

Figure S16. GO enrichment analysis of DEGs in AzaD treated rice plants

Bubble plot shows the GO enrichment results of **(A)** up-regulated and **(B)** down-regulated genes in AzaD treated rice plants. The x axis represents the number of genes in the specific GO item. The y axis represents all the enriched GO items.

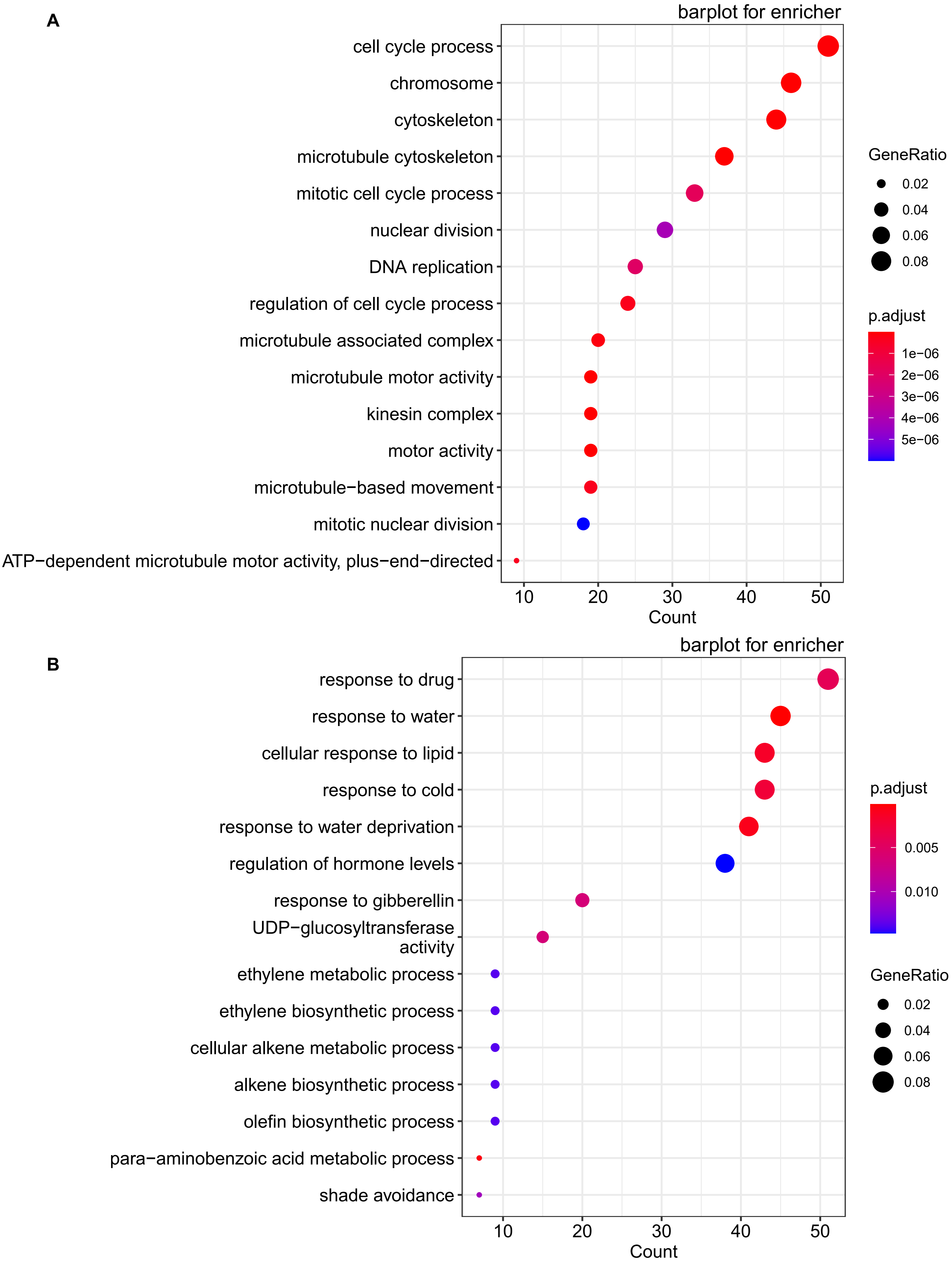

Figure S17. GO enrichment analysis of DEGs in *osmet1-2* mutant rice plants
Bubble plot shows the GO enrichment results of **(A)** up-regulated and **(B)** down-regulated genes in *osmet1-2* mutant rice plants. The x axis represents the number of genes in the specific GO item. The y axis represents all the enriched GO items.

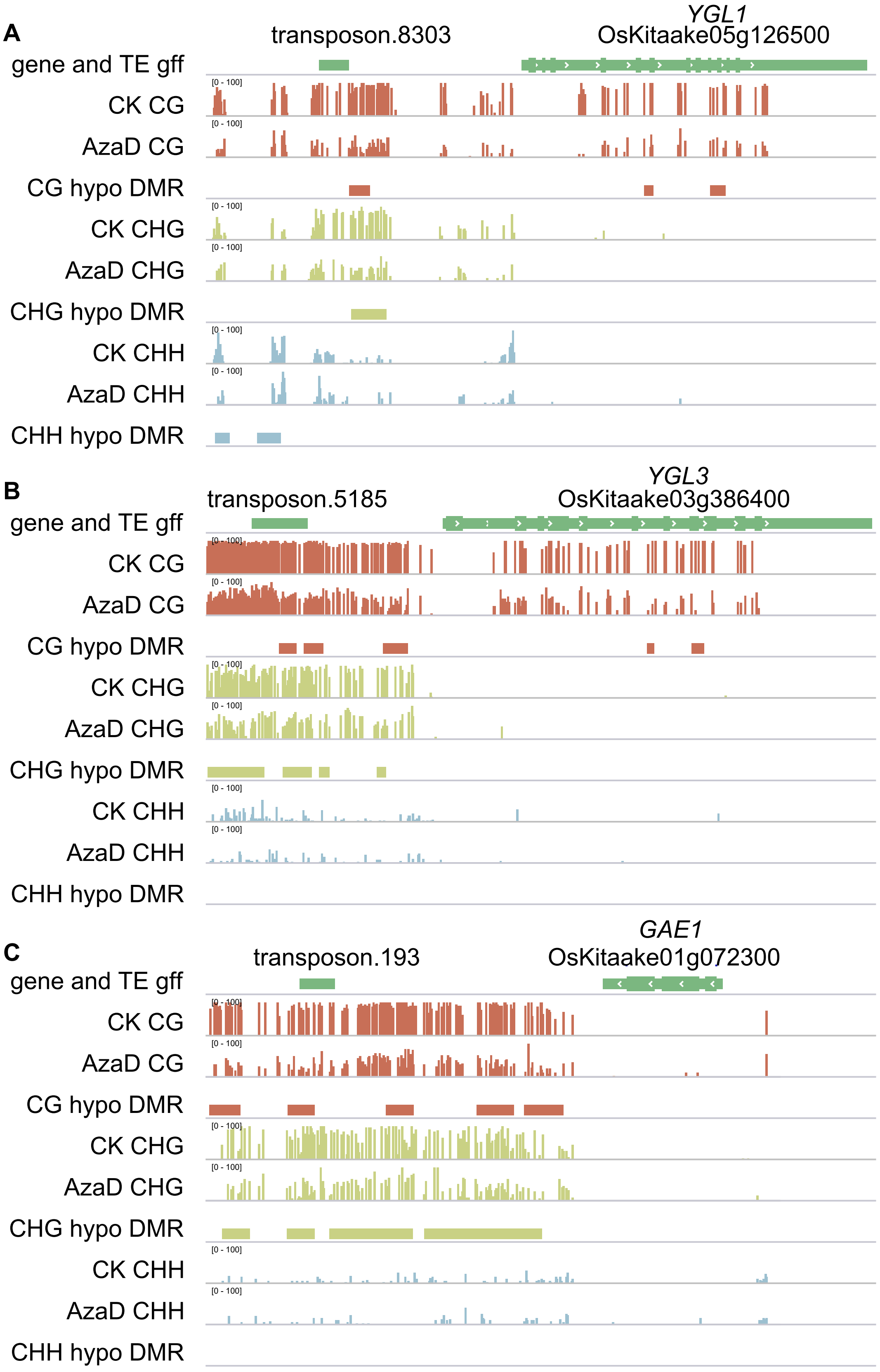

Figure S18. Examples of DNA methylation profiles in three down-regulated genes

IGV screenshots of **(A)** *OsYGL1*, **(B)** *OsYGL3*, and **(C)** *OsGAE1*. TEs, CG methylation, CG hypomethylation regions, CHG methylation, CHG hypomethylation regions, CHH methylation, CHH hypomethylation regions within the flanking regions of the three genes are shown.

Table S1. TE count from different superfamilies in CK and AzaD treatment plants

Superfamily	Expressed	Activated	Not activated	Total(all)	Close	Far
PIF	347	140	207	798	562	236
TcMar	233	86	147	632	441	191
SINE	70	42	28	117	72	45
MuDR	795	474	321	1358	767	591
LINE	621	340	281	2644	1519	1125
hAT	239	179	60	506	266	240
Helitron	101	33	68	345	171	174
Caulimoviru	26	20	6	94	38	56
Copia	911	707	204	2044	744	1300
EnSpm	593	415	178	1573	410	1163
Gypsy	3562	2866	696	7160	1350	5810

Table S2. TE count from different superfamilies in WT and *osmet1-2* mutant plants

Superfamily	Expressed	Activated	Not activated	Total(all)	Close	Far
Mariner	15	6	9	40	32	8
SINE	6	0	6	12	9	3
LINE	25	1	24	111	54	57
Mutator	31	1	30	60	36	24
Pong	43	4	39	109	50	59
FDs	14	4	10	28	13	15
classII_unclassified	757	79	678	1497	665	832
Copia	242	7	235	775	260	515
CACTA	813	30	783	2090	568	1522
classI_unclassified	2226	78	2148	8310	2248	6062
centromere-spe	116	5	111	618	88	530
Gypsy	886	11	875	3714	468	3246

Table S3. PCR primer sets used in RT-qPCR

Gene	Primer Sequence Forward (5'-3')	Primer Sequence Reverse (5'-3')
OsMET1-2	GCGCCAGTAAACTCCTACTT	CCCAATCCAGCCTACCATAAA
OsCMT3	ATCTGCCTGTGCTTGTTCTG	ATCTGCCTGTGCTTGTTCTG
OsCMT2	TCAGGTTGTGGTGGCTTTAC	CTCTGTGTCTTTGGAGGTTGAG
OsDNMT2	GACACCTACATTCCTAACATTGG	TCAGCGACATTCAGACTTATTG
OsDRM2	CGTGCGGCATCTTACTACTGA	ATCTCGGTGATGGCGGTTG
